# Angiotensin-converting enzyme inhibitors, angiotensin receptor blockers, and COVID-19-related outcomes: A patient-level analysis of the PCORnet blood pressure control lab

**DOI:** 10.1016/j.ahjo.2022.100112

**Published:** 2022-03-02

**Authors:** Steven M. Smith, Raj A. Desai, Marta G. Walsh, Ester Kim Nilles, Katie Shaw, Myra Smith, Alanna M. Chamberlain, Catherine G. Derington, Adam P. Bress, Cynthia H. Chuang, Daniel E. Ford, Bradley W. Taylor, Sravani Chandaka, Lav Parshottambhai Patel, James McClay, Elisa Priest, Jyotsna Fuloria, Kruti Doshi, Faraz S. Ahmad, Anthony J. Viera, Madelaine Faulkner, Emily C. O'Brien, Mark J. Pletcher, Rhonda M. Cooper-DeHoff

**Affiliations:** a Department of Pharmacotherapy and Translational Research, College of Pharmacy, University of Florida, Gainesville, FL, United States of America; b Department of Pharmaceutical Outcomes and Policy, College of Pharmacy, University of Florida, Gainesville, FL, United States of America; c Duke Clinical Research Institute, Duke University, Durham, NC, United States of America; d Department of Health Outcomes and Biomedical Informatics, College of Medicine, University of Florida, Gainesville, FL, United States of America; e Departments of Quantitative Health Sciences and Cardiovascular Medicine, Mayo Clinic, Rochester, MN, United States of America; f Department of Population Health Sciences, School of Medicine, University of Utah, Salt Lake City, UT, United States of America; g Penn State University, Hershey, PA, United States of America; h Johns Hopkins University, Baltimore, MD, United States of America; i Medical College of Wisconsin, Milwaukee, WI, United States of America; j University of Kansas Medical Center, Kansas City, KS, United States of America; k University of Nebraska, Omaha, NE, United States of America; l Baylor Scott & White Health, Dallas, TX, United States of America; m School of Medicine, Louisiana State University, New Orleans, LA, United States of America; n Cook County Health, Chicago, IL, United States of America; o Departments of Medicine and Preventive Medicine, Feinberg School of Medicine, Northwestern University, Chicago, IL, United States of America; p Department of Family Medicine and Community Health, School of Medicine, Duke University, Durham, NC, United States of America; q Department of Epidemiology & Biostatistics, School of Medicine, University of California San Francisco, San Francisco, CA, United States of America

**Keywords:** Covid-19, ACE inhibitors, ARBs, PCORnet, Hypertension

## Abstract

SARS-CoV-2 accesses host cells via angiotensin-converting enzyme-2, which is also affected by commonly used angiotensin-converting enzyme inhibitors (ACEIs) and angiotensin receptor blockers (ARBs), raising concerns that ACEI or ARB exposure may portend differential COVID-19 outcomes. In parallel cohort studies of outpatient and inpatient COVID-19-diagnosed adults with hypertension, we assessed associations between antihypertensive exposure (ACEI/ARB vs. non-ACEI/ARB antihypertensives, as well as between ACEI- vs. ARB) at the time of COVID-19 diagnosis, using electronic health record data from PCORnet health systems. The primary outcomes were all-cause hospitalization or death (outpatient cohort) or all-cause death (inpatient), analyzed via Cox regression weighted by inverse probability of treatment weights. From February 2020 through December 9, 2020, 11,246 patients (3477 person-years) and 2200 patients (777 person-years) were included from 17 health systems in outpatient and inpatient cohorts, respectively. There were 1015 all-cause hospitalization or deaths in the outpatient cohort (incidence, 29.2 events per 100 person-years), with no significant difference by ACEI/ARB use (adjusted HR 1.01; 95% CI 0.88, 1.15). In the inpatient cohort, there were 218 all-cause deaths (incidence, 28.1 per 100 person-years) and ACEI/ARB exposure was associated with reduced death (adjusted HR, 0.76; 95% CI, 0.57, 0.99). ACEI, versus ARB exposure, was associated with higher risk of hospitalization in the outpatient cohort, but no difference in all-cause death in either cohort. There was no evidence of effect modification across pre-specified baseline characteristics. Our results suggest ACEI and ARB exposure have no detrimental effect on hospitalizations and may reduce death among hypertensive patients diagnosed with COVID-19.

## 1 Introduction

The severe acute respiratory syndrome coronavirus 2 (SARS-CoV-2), responsible for the Coronavirus Disease-2019 (COVID-19) pandemic, accesses human host cells via angiotensin-converting enzyme 2 (ACE2) [1], [2]. An integral component of the renin angiotensin aldosterone system (RAAS), ACE2 is responsible for conversion of angiotensin II to angiotensin (1–7), a potent vasodilator and anti-inflammatory compound, which counteracts the vasoconstrictor and inflammatory effects of angiotensin II. The RAAS is a common target of cardiovascular pharmacotherapy, particularly with angiotensin-converting enzyme inhibitors (ACEIs) and angiotensin receptor blockers (ARBs), two of the most commonly prescribed drug classes in the U.S. and globally. Accordingly, following the discovery of SARS-CoV-2 mechanism of entry into host cells (i.e., ACE2), substantial interest emerged concerning whether exposure to ACEIs, ARBs or both may be protective or detrimental for patients infected by SARS-CoV-2.

Recent randomized controlled trials testing continuation vs. discontinuation of ACEI/ARB therapy have suggested no differential risk of infection or COVID-19 severity between these strategies [3], [4]. Results from the observational studies have been much more variable, with some suggesting substantially lower mortality (~50–60% risk reductions) for ACEI/ARB users vs. non-users [5], [6], [7], whereas others have suggested higher risk of mortality [8]. However, many of these studies have significant methodologic limitations [9], and are limited by their homogenous populations, making interpretation and generalizability difficult. While the most robust observational studies seem to suggest no increased risk, or possibly more modest benefits, from ACEI/ARB exposure, these have been mostly limited to homogenous populations.

Accordingly, to overcome limitations to the existing data and expand the generalizability to diverse populations, we tested associations between COVID-19 and ACEI/ARB exposure in parallel cohort studies of COVID-19-diagnosed outpatients and inpatients using patient-level data from a geographically and racially-diverse patient population from 17 health system partners in the U.S.-based National Patient-Centered Clinical Research Network (PCORnet). We further sought to test and replicate prior findings that ARBs may be protective against COVID-19 severity compared with ACEIs [10] given that these classes are known to affect ACE2 expression differentially across organs [11], [12].

## 2 Methods

We conducted retrospective cohort studies using patient-level electronic health record (EHR) data from health systems in the PCORnet Blood Pressure Control Laboratory (BP Control Lab) who agreed to participate in this patient-level analysis. The University of Florida (UF) served as the data core for this study and the Institutional Review Board at each health system approved the study with waivers of informed consent.

### 2.1 Data sources

The BP Control Lab is an established research collaboration, including 27 health systems, that leverages PCORnet infrastructure, including the PCORnet common data model (CDM), to support large-scale observational studies and national surveillance, large pragmatic RCTs, and local quality improvement efforts centered on hypertension and related cardiovascular disease [13], [14]. The PCORnet CDM facilitates standardization of EHR data, including patient demographics, encounters, diagnoses, procedures, medications (prescribed and dispensed), vitals, laboratory measures, and related domains. Health systems participating in PCORnet undergo quarterly data characterization by the PCORnet data coordinating center (DCC) at Duke University to ensure minimum data quality standards and certification of research-ready data. For the present study, a data query was developed by the BP Control Lab data core at UF, in collaboration with the PCORnet DCC, and distributed to participating BP Control Lab health systems (Supplemental Table S1). Patient-level data were returned to the PCORnet DCC for data quality checking and subsequently transmitted to the UF data core for analysis.

### 2.2 Cohort development

We developed separate, mutually exclusive cohorts for individuals diagnosed with COVID-19 initially in the outpatient and inpatient settings. Cohort design schematics are presented in Supplemental Figs. S1 and S2. Briefly, eligible patients were patients aged ≥18 years, with a first COVID-19 diagnosis (ICD-CM-10, U07.1) in the outpatient (outpatient cohort) or inpatient (inpatient cohort) setting on or after February 1, 2020 through December 9, 2020; patients with a first COVID-19 diagnosis in both settings on the same day were included in the inpatient cohort only. These diagnostic codes have been shown to have high sensitivity and PPV in hospitalized patients [15]. Encounters were defined as inpatient or outpatient using CPT evaluation and management codes (Supplemental Table S2). The date of first COVID-19 diagnosis was considered the index date. Patients were also required to have ≥1 prescription or dispensing of an antihypertensive drug (Supplemental Table S3) prescribed or filled within the year prior to (and excluding) the index date. Patients were excluded if they lacked a hypertension diagnosis (Supplemental Table S4) during the year prior to and including the index date. To minimize inclusion of patients who were not routine users of the health system in which they received a COVID-19 diagnosis, we excluded, from both cohorts, individuals lacking ≥2 encounters of any type within the same health system in the 2 years preceding the index date. For the outpatient cohort, we further excluded individuals with a hospitalization in the 30 days prior to the index date.

### 2.3 Exposures

In both cohorts, we assigned patients to exposure groups based on antihypertensive medication use in the 365 days prior to, and excluding, the index date. Patients receiving any prescription or dispensing for ≥1 ACE inhibitor or ARB, irrespective of other antihypertensive use, were considered ACEI-/ARB-exposed; all other patients (all of whom were, by definition, treated with ≥1 antihypertensive) were considered non-ACEI-/ARB antihypertensive-exposed. In secondary analyses, comparing ACEI versus ARB exposure, we excluded individuals exposed to both ≥1 ACEI and ≥1 ARB during the year prior to the index date.

### 2.4 Outcomes

Supplemental Table S5 summarizes measurement approaches for all study outcomes. The primary outcome for the outpatient cohort was first occurrence of all-cause hospitalization or all-cause death, with each analyzed separately as secondary outcomes. The primary outcome for the inpatient cohort was all-cause death. Exploratory secondary outcomes in the inpatient cohort included ICU admission, mechanical ventilation, and dialysis during the index hospital stay. For primary outcomes, patients without an outcome were censored on the last encounter date observed for the respective health system, or the date on which the query was distributed by the DCC (December 9, 2020), whichever came first. For mechanical ventilation, ICU admission, and dialysis, patients were censored at discharge (from the index hospitalization) or, absent a discharge date, the last encounter date (from the respective health system) or December 9, 2020, whichever came first.

### 2.5 Covariates

Data on demographics, comorbidities, vital signs, laboratory measurements and concomitant medications were collected at baseline using data on, or within the 1 year preceding, the index date, unless otherwise noted in Supplemental Table S6. Clinical measurements on or closest to the index date were prioritized. Multiple imputation (*n* = 10 imputations) was used to address missingness among clinical measurements.

### 2.6 Propensity score

Separately for each cohort, we developed multivariable logistic regression models to estimate probabilities (i.e., a propensity score [PS]) for being ACEI/ARB-exposed versus non-ACEI/ARB-exposed, as well as ACEI- versus ARB-exposed. Models were generated for each imputed dataset of each cohort. All baseline covariates (Supplemental Table S6) were included as independent variables. Common support regions were examined comparing histograms across exposures. The PS was used to calculate inverse probability of treatment weights (IPTWs) for the primary analysis. Covariate balance was verified in the IPTW-weighted and matched populations via absolute standardized mean differences, with ≤0.1 considered well-balanced.

### 2.7 Statistical analyses

Analyses were performed separately for each cohort. Crude event rates were calculated as number of events per 100 person-years. Proportional hazards regression models were fit for each outcome, weighted by the IPTW for the primary analysis. Sensitivity analyses included proportional hazards models developed in the 1:1 matched cohorts. In each case, separate models were generated for each imputed dataset, and results were then combined according to Rubin's rules [16]. For ACEI/ARB vs. non-ACEI/ARB exposure comparisons only, we also conducted sensitivity analyses excluding individuals with diabetes, coronary heart disease, kidney disease, heart failure with reduced ejection fraction, or stroke (“compelling indications” for ACEI/ARB therapy) to explore potential for confounding by indication. Secondary analyses were performed for the primary outcomes, with results stratified by age, sex, race/ethnicity, BMI category, and systolic and diastolic BP categories to explore potential effect modification. Negative control analyses were performed to assess residual confounding. For both cohorts negative control outcomes were gastrointestinal bleeding and urinary tract infection (Supplemental Table S5), neither known to be associated with specific antihypertensive agents. A two-sided α = 0.05 was used for all hypothesis testing and without correction for multiple comparisons. All analyses were performed in SAS 9.4 (SAS Institute, Cary, NC, USA).

## 3 Results

Among patients first diagnosed with COVID-19 in the outpatient setting, 11,246 patients from the 17 participating health systems met eligibility criteria, including 3583 (32%) non-ACEI/ARB antihypertensive-exposed and 7663 (68%) ACEI- or ARB-exposed (Supplemental Fig. S3). Of the ACEI/ARB-exposed, 3838 (50%) were exposed to ACEIs only and 3612 (47%) to ARBs only; 213 (3%) had ACEI and ARB exposure during the baseline period and were excluded from the ACEI vs. ARB comparisons. Baseline characteristics for these individuals are summarized in Table 1 (ACEI/ARB vs. non-ACEI/ARB exposed) and Supplemental Table S7 (ACEI vs. ARB exposed).

**Table 1:** Baseline characteristics of the outpatient COVID-19 cohort.

Baseline characteristic	Overall cohort (n = 11,246)	ACEI/ARB exposed (n = 7663)	non-ACEI/ARB exposed (n = 3583)
Demographics			
Age, years	61.2 ± 12.7	61.6 ± 12.2	60.5 ± 13.5
<45	1140 (10%)	683 (9%)	457 (13%)
45–64	5426 (48%)	3736 (49%)	1690 (47%)
≥65	4680 (42%)	3244 (42%)	1436 (40%)
Sex			
Female	6262 (56%)	4081 (53%)	2181 (61%)
Male	4983 (44%)	3581 (47%)	1402 (39%)
Unknown	1 (0%)	1 (0%)	0 (0%)
Race, self-reported			
American Indian or Alaska Native	68 (1%)	52 (1%)	16 (0%)
Asian	286 (3%)	215 (3%)	71 (2%)
Black or African American	3059 (27%)	1888 (25%)	1171 (33%)
Native Hawaiian or Other Pacific Islander	40 (0%)	29 (0%)	11 (0%)
White	6231 (55%)	4365 (57%)	1866 (52%)
Multiple race	81 (1%)	55 (1%)	26 (1%)
Ethnicity			
Non-Hispanic	8956 (80%)	6010 (78%)	2946 (82%)
Hispanic	1716 (15%)	1258 (16%)	458 (13%)
Height, inches	66.3 ± 4.2	66.4 ± 4.3	66.1 ± 4.1
Missing data	1877 (17%)	1268 (17%)	609 (17%)
Weight, lbs	192.3 ± 1.9	192.3 ± 1.9	192.3 ± 1.9
Missing data	9888 (88%)	6745 (88%)	3143 (88%)
Body mass index, kg/m^2^	32.7 ± 8.0	33.0 ± 8.0	32.2 ± 8.0
Missing data	4787 (43%)	3314 (43%)	1473 (41%)
Vitals & labs			
Blood pressure, mm Hg			
Systolic	133 ± 18	133 ± 18	132 ± 17
Diastolic	78 ± 11	78 ± 11	78 ± 11
Missing BP data	3695 (33%)	2517 (33%)	1178 (33%)
Total cholesterol, mg/dL	172 ± 45	170 ± 46	177 ± 44
Missing data	4204 (37%)	2706 (35%)	1498 (42%)
HDL-C, mg/dL	51 ± 15	50 ± 15	52 ± 16
Missing data	4907 (44%)	3201 (42%)	1706 (48%)
LDL-C, mg/dL	96 ± 36	95 ± 36	100 ± 36
Missing data	4339 (39%)	2799 (37%)	1540 (43%)
Triglyceride, mg/dL	142 ± 98	144 ± 103	135 ± 83
Missing data	4450 (40%)	2875 (38%)	1575 (44%)
Hemoglobin A1c, %	6.74 ± 1.66	6.86 ± 1.71	6.41 ± 1.48
Missing data	5065 (45%)	3195 (42%)	1870 (52%)
Serum creatinine, mg/dL	1.01 ± 0.48	1.00 ± 0.44	1.02 ± 0.56
Missing data	1922 (17%)	1280 (17%)	642 (18%)
Estimated GFR, mL/min/1.73m^2^	73.58 ± 25.28	73.62 ± 24.89	73.50 ± 26.12
Missing data	5667 (50%)	3855 (50%)	1812 (51%)
Serum potassium, mg/dL	4.21 ± 0.48	4.23 ± 0.47	4.17 ± 0.50
Missing data	2368 (21%)	1554 (20%)	814 (23%)
Comorbidities			
Current smoking	1682 (15%)	1114 (15%)	568 (16%)
Diabetes	4642 (41%)	3561 (46%)	1081 (30%)
Chronic kidney disease	2782 (25%)	1958 (26%)	824 (23%)
End-stage renal disease	6 (0%)	3 (0%)	3 (0%)
History of kidney transplant	10 (0%)	6 (0%)	4 (0%)
Heart failure with reduced EF	599 (5%)	387 (5%)	212 (6%)
History of CHD	1681 (15%)	1169 (15%)	512 (14%)
Prior coronary revascularization	132 (1%)	91 (1%)	41 (1%)
History of stroke	365 (3%)	266 (3%)	99 (3%)
History of PAD	306 (3%)	212 (3%)	94 (3%)
History of ASCVD	2055 (18%)	1443 (19%)	612 (17%)
Atrial fibrillation	660 (6%)	403 (5%)	257 (7%)
Chronic obstructive pulmonary disease	787 (7%)	489 (6%)	298 (8%)
Asthma	1361 (12%)	906 (12%)	455 (13%)
History of depression	1754 (16%)	1191 (16%)	563 (16%)
Charlson Comorbidity Score	2.40 ± 3.26	2.29 ± 3.12	2.62 ± 3.52
Medication use			
Statin	3085 (27%)	2297 (30%)	788 (22%)
Aspirin	1058 (9%)	754 (10%)	304 (8%)
Anticoagulants	926 (8%)	615 (8%)	311 (9%)
Antihypertensives			
ACE inhibitor	4051 (36%)	4051 (53%)	0 (0%)
ARB	3825 (34%)	3825 (50%)	0 (0%)
Direct renin inhibitor	6 (0%)	1 (0%)	5 (0%)
Aldosterone receptor antagonist	550 (5%)	337 (4%)	213 (6%)
Dihydropyridine CCB	3946 (35%)	2397 (31%)	1549 (43%)
Non-dihydropyridine CCB	490 (4%)	287 (4%)	203 (6%)
Thiazide diuretic	4195 (37%)	3015 (39%)	1180 (33%)
Loop diuretic	1391 (12%)	912 (12%)	479 (13%)
Potassium-sparing diuretic	306 (3%)	150 (2%)	156 (4%)
β-blocker	4485 (40%)	2714 (35%)	1771 (49%)
α_1_ blocker	224 (2%)	152 (2%)	72 (2%)
α_2_ agonist	240 (2%)	149 (2%)	91 (3%)
Direct vasodilator	493 (4%)	338 (4%)	155 (4%)
Insurance type			
Medicaid	494 (4%)	317 (4%)	177 (5%)
Medicare	1571 (14%)	1096 (14%)	475 (13%)
Other government	191 (2%)	139 (2%)	52 (1%)
Commercial insurance or managed care	1975 (18%)	1328 (17%)	647 (18%)
Self-pay or charity care	164 (1%)	117 (2%)	47 (1%)
Other	67 (1%)	45 (1%)	22 (1%)
Unknown	891 (8%)	639 (8%)	252 (7%)
Missing data	5893 (52%)	3982 (52%)	1911 (53%)

ACEI, angiotensin-converting enzyme inhibitor; ARB, angiotensin receptor blocker; ASCVD, atherosclerotic cardiovascular disease; CCB, calcium channel blocker; CHD, coronary heart disease; COPD, chronic obstructive pulmonary disease; EF, ejection fraction; GFR, glomerular filtration rate; HDL—C, high-density lipoprotein cholesterol; LDL-C, low-density lipoprotein cholesterol; PAD, peripheral arterial disease.

Among patients first diagnosed with COVID-19 in the inpatient setting, 2200 met eligibility criteria, including 737 (34%) non-ACEI/ARB antihypertensive-exposed and 1463 (67%) ACEI- or ARB-exposed (Supplemental Fig. S4). Among those ACEI/ARB-exposed, 790 (54%) were exposed to ACEIs only and 617 (42%) to ARBs only; 56 (4%) were exposed to both and were excluded from ACEI vs. ARB comparisons. Baseline characteristics of these patients are summarized in Tables 2 and Supplemental Table S8.

**Table 2:** Baseline characteristics of the inpatient COVID-19 cohort.

Baseline characteristic	Overall cohort (*n* = 2200)	ACEI/ARB exposed (*n* = 1463)	non-ACEI/ARB exposed (*n* = 737)
Demographics			
Age, years	66.6 ± 12.6	66.1 ± 12.1	67.5 ± 13.5
<45	127 (6%)	76 (5%)	51 (7%)
45–64	741 (34%)	531 (36%)	210 (28%)
≥65	1332 (61%)	856 (59%)	476 (65%)
Sex			
Female	1110 (50%)	727 (50%)	383 (52%)
Male	1090 (50%)	736 (50%)	354 (48%)
Unknown	0 (0%)	0 (0%)	0 (0%)
Race, self-reported			
American Indian or Alaska Native	26 (1%)	19 (1%)	7 (1%)
Asian	47 (2%)	33 (2%)	14 (2%)
Black or African American	677 (31%)	448 (31%)	229 (31%)
Native Hawaiian or Other Pacific Islander	11 (1%)	6 (0%)	5 (1%)
White	1204 (55%)	782 (53%)	422 (57%)
Multiple races	15 (1%)	11 (1%)	4 (1%)
Ethnicity			
Non-Hispanic	1896 (86%)	1248 (85%)	648 (88%)
Hispanic	269 (12%)	196 (13%)	73 (10%)
Height, inches	66.7 ± 4.3	66.8 ± 4.3	66.3 ± 4.4
Missing data	168 (8%)	104 (7%)	64 (9%)
Weight, pounds	192.1 ± 1.9	192.1 ± 1.9	192.2 ± 2.0
Missing data	1851 (84%)	1220 (83%)	631 (86%)
Body mass index, kg/m^2^	32.6 ± 8.6	32.6 ± 8.2	32.5 ± 9.2
Missing data	678 (31%)	446 (30%)	232 (31%)
Vitals & Labs			
Blood pressure, mm Hg			
Systolic	132 ± 20	133 ± 21	129 ± 19
Diastolic	75 ± 12	76 ± 13	75 ± 12
Missing BP data	764 (35%)	520 (36%)	244 (33%)
Total cholesterol, mg/dL	160 ± 48	159 ± 48	164 ± 46
Missing data	1035 (47%)	625 (43%)	410 (56%)
HDL-C, mg/dL	47 ± 15	47 ± 15	49 ± 15
Missing data	1054 (48%)	640 (44%)	414 (56%)
LDL-C, mg/dL	87 ± 37	85 ± 38	92 ± 35
Missing data	1050 (48%)	637 (44%)	413 (56%)
Triglyceride, mg/dL	144 ± 88	149 ± 89	132 ± 84
Missing data	1022 (46%)	622 (43%)	400 (54%)
Hemoglobin A1c, %	7.17 ± 1.90	7.32 ± 1.95	6.79 ± 1.71
Missing data	960 (44%)	581 (40%)	379 (51%)
Serum creatinine, mg/dL	1.29 ± 1.10	1.32 ± 1.23	1.22 ± 0.78
Missing data	233 (11%)	158 (11%)	75 (10%)
Estimated GFR, mL/min/1.73m^2^	53.05 ± 25.53	52.58 ± 25.57	54.00 ± 25.46
Missing data	820 (37%)	543 (37%)	277 (38%)
Serum potassium, mg/dL	4.15 ± 0.58	4.16 ± 0.58	4.12 ± 0.57
Missing data	277 (13%)	192 (13%)	85 (12%)
Comorbidities			
Current smoking	775 (35%)	502 (34%)	273 (37%)
Diabetes	1285 (58%)	928 (63%)	357 (48%)
Chronic kidney disease	1227 (56%)	825 (56%)	402 (55%)
End-stage renal disease	44 (2%)	28 (2%)	16 (2%)
History of kidney transplant	7 (0%)	2 (0%)	5 (1%)
Heart failure with reduced EF	437 (20%)	293 (20%)	144 (20%)
History of CHD	766 (35%)	502 (34%)	264 (36%)
Prior coronary revascularization	48 (2%)	34 (2%)	14 (2%)
History of stroke	206 (9%)	142 (10%)	64 (9%)
History of PAD	224 (10%)	141 (10%)	83 (11%)
History of ASCVD	939 (43%)	623 (43%)	316 (43%)
Atrial fibrillation	392 (18%)	225 (15%)	167 (23%)
COPD	536 (24%)	336 (23%)	200 (27%)
Asthma	421 (19%)	282 (19%)	139 (19%)
History of depression	681 (31%)	435 (30%)	246 (33%)
Charlson Comorbidity Score	6.44 ± 4.25	6.27 ± 4.16	6.76 ± 4.39
Medication use			
Statin	875 (40%)	635 (43%)	240 (33%)
Aspirin	483 (22%)	341 (23%)	142 (19%)
Anticoagulants	546 (25%)	344 (24%)	202 (27%)
Antihypertensives			
ACE inhibitor	846 (38%)	846 (58%)	0 (0%)
ARB	673 (31%)	673 (46%)	0 (0%)
Direct renin inhibitor	1 (0%)	1 (0%)	0 (0%)
Aldosterone receptor antagonist	219 (10%)	144 (10%)	75 (10%)
Dihydropyridine CCB	888 (40%)	587 (40%)	301 (41%)
Non-dihydropyridine CCB	165 (8%)	100 (7%)	65 (9%)
Thiazide diuretic	677 (31%)	516 (35%)	161 (22%)
Loop diuretic	715 (33%)	448 (31%)	267 (36%)
Potassium-sparing diuretic	42 (2%)	22 (2%)	20 (3%)
β-blocker	1245 (57%)	780 (53%)	465 (63%)
α_1_ blocker	67 (3%)	43 (3%)	24 (3%)
α_2_ agonist	85 (4%)	58 (4%)	27 (4%)
Direct vasodilator	294 (13%)	196 (13%)	98 (13%)
Insurance type			
Medicaid	170 (8%)	125 (9%)	45 (6%)
Medicare	628 (29%)	406 (28%)	222 (30%)
Other government	32 (1%)	20 (1%)	12 (2%)
Commercial Insurance or Managed Care	242 (11%)	177 (12%)	65 (9%)
Self-pay or charity care	27 (1%)	22 (2%)	5 (1%)
Other	26 (1%)	17 (1%)	9 (1%)
Unknown	75 (3%)	52 (4%)	23 (3%)
Missing data	1000 (45%)	644 (44%)	356 (48%)

ACEI, angiotensin-converting enzyme inhibitor; ARB, angiotensin receptor blocker; ASCVD, atherosclerotic cardiovascular disease; CCB, calcium channel blocker; CHD, coronary heart disease; COPD, chronic obstructive pulmonary disease; EF, ejection fraction; GFR, glomerular filtration rate; HDL—C, high-density lipoprotein cholesterol; LDL-C, low-density lipoprotein cholesterol; PAD, peripheral arterial disease.

In both cohorts, the majority of patients were women, just over half were white and most were non-Hispanic, though significant proportions of each cohort comprised racial minorities. Most patients were aged ≥60 years, particularly in the inpatient cohort, and substantial proportions had a history of diabetes (41% in outpatient cohort; 58% in inpatient cohort), with significantly higher proportions among ACEI or ARB users; a history of ASCVD, depression, and chronic kidney disease were also common across both cohorts, though with only modest differences observed between exposure groups. Among non-ACEI/ARB-exposed, primary antihypertensive use consisted of β-blockers, thiazide diuretics, and/or dihydropyridine calcium channel blockers. After weighting, we observed no significant differences (i.e., all absolute standardized mean differences <0.1) in baseline characteristics between comparison groups (Supplemental Fig. S5).

### 3.1 Outcomes

**Fig. 1:**
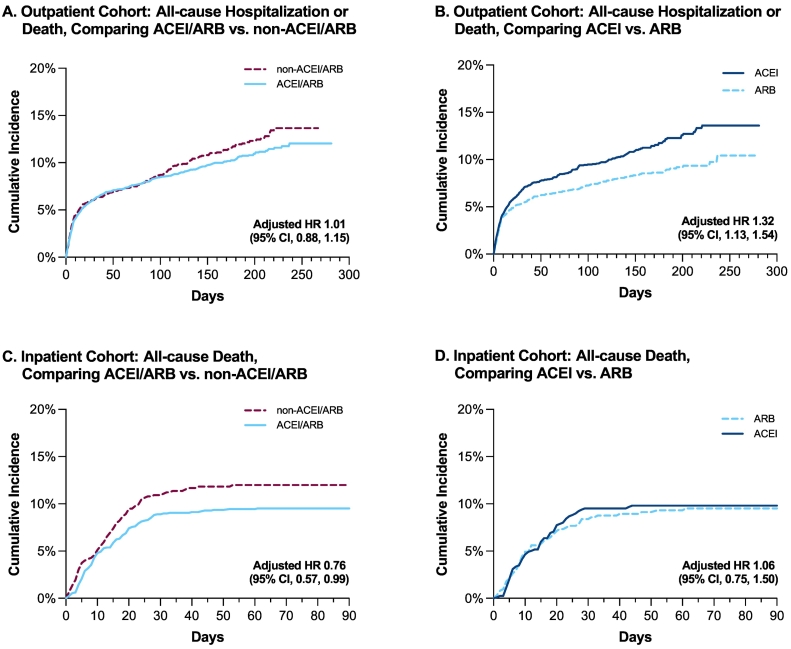
Cumulative incidence of the primary outcomes in the outpatient (panels A and B) and inpatient (panels C and D) cohorts.

Numbers of events, incidence rates, and crude and adjusted hazard ratios for the outpatient cohort are presented in Table 3. Briefly, there were a total of 1015 all-cause hospitalizations or all-cause death outcomes over a cumulative 3477 person-years (29.2 per 100 person-years). The crude incidence rate for ACEI/ARB-exposed was 28.6 per 100 person-years versus 30.5 per 100 person-years for non-ACEI/ARB-exposed, with a crude hazard ratio of 0.92 (95% CI, 0.81, 1.05). After IPTW-weighting, there was no significant association between ACEI/ARB exposure and the primary outcome (adjusted HR, 1.01; 95% CI, 0.88, 1.15) (Fig. 1). Results were qualitatively similar for all-cause death and hospitalization, analyzed separately (Table 3).

**Table 3:** Incidence rates and hazard ratios for primary and secondary outcomes in the outpatient cohort.

Outcome	ACEI/ARB- vs. non-ACEI/ARB-exposed analysis		ACEI vs. ARB-exposed analysis	
	ACEI/ARB-exposed	Non-ACEI/ARB-exposed	ACEI-exposed	ARB-exposed
Primary outcome				
All-cause hospitalization or all-cause death				
No. of events	671	344	373	274
Person-time⁎	2349	1128	1140	1143
Rate†	28.6	30.5	32.7	24.0
Crude HR (95% CI)	0.92 (0.81, 1.05)	Ref.	1.32 (1.13, 1.54)	Ref.
Adjusted HR (95% CI)	1.01 (0.88, 1.15)	Ref.	1.32 (1.13, 1.54)	Ref.
Secondary outcomes				
All-cause death				
No. of events	106	62	54	48
Person-time⁎	2511	1205	1230	1211
Rate†	4.2	5.1	4.4	4.0
Crude HR (95% CI)	0.80 (0.59, 1.10)	Ref.	1.07 (0.72, 1.57)	Ref.
Adjusted HR (95% CI)	0.78 (0.58, 1.07)	Ref.	1.14 (0.78, 1.68)	Ref.
All-cause hospitalization				
No. of events	565	282	319	226
Person-time⁎	2407	1160	1169	1170
Rate†	23.5	24.3	27.3	19.4
Crude HR (95% CI)	0.95 (0.82, 1.10)	Ref.	1.37 (1.16, 1.63)	Ref.
Adjusted HR (95% CI)	1.07 (0.92, 1.24)	Ref.	1.35 (1.14, 1.61)	Ref.

⁎ Cumulative person-years (sum of all time-to-event across all patients).

† No. of events divided by person-years, expressed per 100 person-years.

Table 4 summarizes outcome data in the inpatient cohort. In sum, there were 218 deaths over 777 cumulative person-years (28.1 per 100 person-years) in the inpatient cohort. The death rate in the ACEI/ARB-exposed group was moderately lower (25.3 per 100 person-years) compared with the non-ACEI/ARB-exposed group (33.9 per 100 person-years). In IPTW-weighted analyses, ACEI/ARB exposure was associated with a 24% reduced risk of all-cause death (adjusted HR, 0.76; 95% CI, 0.57, 0.99). A total of 715 (32.5%) patients had an ICU admission (83 per 100 person-years), 315 (14.3%) received mechanical ventilation (36.8 per 100 person-years), and 52 (2.3%) had incident dialysis (6.1 per 100 person-years). No differences were observed between ACEI/ARB- and non-ACEI/ARB-exposed groups for any of these outcomes in the unadjusted or adjusted analyses.

**Table 4:** Incidence rates and hazard ratios for primary and secondary outcomes in the inpatient cohort.

Outcome	ACEI/ARB- vs. non-ACEI/ARB-exposed analysis		ACEI vs. ARB-exposed analysis	
	ACEI/ARB-exposed	Non-ACEI/ARB-exposed	ACEI-exposed	ARB-exposed
Primary outcome				
All-cause death				
No. of events	133	85	74	56
Person-time⁎	526	251	278	229
Rate†	25.3	33.9	26.7	24.5
Crude HR (95% CI)	0.78 (0.60, 1.03)	Ref.	1.04 (0.73, 1.47)	Ref.
Adjusted HR (95% CI)	0.76 (0.57, 0.99)	Ref.	1.06 (0.75, 1.50)	Ref.
Secondary outcomes				
ICU admission				
No. of events	490	225	278	192
Person-time⁎	581	287	309	250
Rate†	84.4	78.3	89.9	76.7
Crude HR (95% CI)	0.96 (0.82,1.13)	Ref.	1.13 (0.94,1.37)	Ref.
Adjusted HR (95% CI)	0.94 (0.80,1.11)	Ref.	1.07 (0.89,1.29)	Ref.
Mechanical ventilation				
No. of events	223	92	122	93
Person-time⁎	581	287	309	250
Rate†	38.4	32.0	39.4	37.2
Crude HR (95% CI)	1.05 (0.82,1.34)	Ref.	1.00 (0.76,1.31)	Ref.
Adjusted HR (95% CI)	0.97 (0.76,1.24)	Ref.	0.97 (0.74,1.28)	Ref.
Dialysis				
No. of events	39	13	16	22
Person-time⁎	581	287	309	250
Rate†	6.7	4.5	5.2	8.8
Crude HR (95% CI)	1.33 (0.71,2.51)	Ref.	0.52 (0.27,1.00)	Ref.
Adjusted HR (95% CI)	1.19 (0.63,2.25)	Ref.	0.44 (0.22,0.88)	Ref.

⁎ Cumulative person-years (sum of all time-to-event across all patients).

† No. of events divided by person-time, expressed per 100 person-years.

**Fig. 2:**
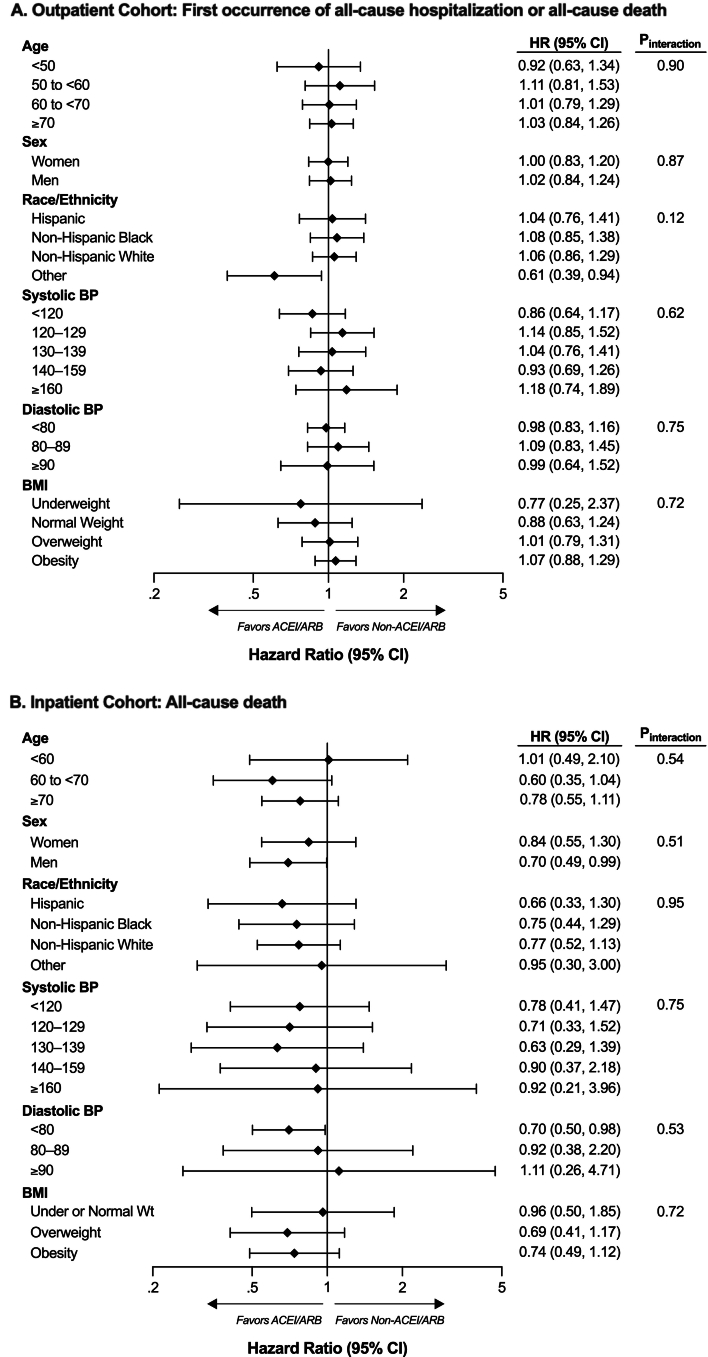
Stratified analyses of the primary outcome in the outpatient (panel A) and inpatient (panel B) cohorts.

In stratified analyses, we observed no significant differences in the primary outcome for either the outpatient or inpatient cohorts across pre-specified baseline characteristics, including age, sex, race/ethnicity, baseline BP categories, or BMI categories (Fig. 2).

Comparing ACEI-exposed vs. ARB-exposed, we observed a significant association between ACEI exposure and higher risk of all-cause death or hospitalization (adjusted HR, 1.32; 95% CI, 1.13, 1.54), primarily driven by a higher risk of all-cause hospitalization (adjusted HR, 1.35; 95% CI, 1.14, 1.61), whereas no difference was observed in all-cause death (adjusted HR, 1.14; 95% CI, 0.78, 1.68). In the inpatient cohort, no difference was observed between ACEI vs. ARB exposure on all-cause death (adjusted HR, 1.06; 95% CI, 0.75, 1.50). Among the secondary outcomes, ACEI exposure was associated only with lower risk of dialysis (Table 3).

### 3.2 Sensitivity analyses

Negative control analyses did not reveal meaningfully different baseline risk across treatment groups. Specifically, we observed no association between ACEI/ARB exposure (vs. non-ACEI/ARB exposure) and GI bleeding or urinary tract infection in both cohorts (Supplemental Table S9), though GI bleeds were rare in the outpatient cohort. Similar results were observed comparing ACEI vs. ARB exposure. Sensitivity analyses using a propensity score matched design revealed qualitatively similar results for the primary outcomes in both cohorts (Supplemental Table S10). Likewise, sensitivity analyses excluding individuals with compelling indications from ACEI/ARB versus non-ACEI/ARB comparisons revealed results similar to the primary analysis for the outpatient cohort (Supplemental Table S11); event rates were low in the inpatient cohort, with substantial imprecision in hazard ratio estimates.

## 4 Discussion

In this large, racially- and geographically-diverse population of U.S. adults with hypertension and diagnosed COVID-19 in 2020, we found no adverse association between prior ACEI or ARB exposure and COVID-19, regardless of whether the patient was first diagnosed in the inpatient or outpatient setting. Specifically, we observed no difference in all-cause hospitalizations or death, either as a composite outcome or individually, comparing ACEI/ARB-exposed versus other antihypertensive exposures in patients with COVID-19 in the outpatient setting. These results were robust across several sensitivity analyses, even when restricting the analysis to individuals without compelling indications for ACEI/ARB therapy. We also found evidence of a possible mortality benefit (24% lower risk) associated with ACEI/ARB exposure in the inpatient setting in our primary analysis (IPTW-weighted). In sensitivity analyses, using a PS-matched approach rather than IPTW-weighting, we observed a similar point estimate (~25% lower mortality risk with ACEI/ARB exposure), though the confidence interval included 1. Finally, we observed a significantly lower risk of hospitalization among ARB-exposed individuals with COVID-19, compared with similar ACE-I-exposed individuals. Taken together, these results are consistent with recent clinical trial findings and high-quality observational studies, and they expand these to a larger and considerably more diverse U.S. population, allowing us to test effect heterogeneity across important demographic and clinical strata. Overall, this analysis broadly supports existing recommendations to continue ACEI/ARB therapy in patients with indications for such therapy.

Hypertension is now a well-known risk factor for COVID-19 severity, yet early epidemiologic studies raised questions about whether hypertension per se, or possibly some of its treatments, might be responsible for the 1–3-fold increases in morbidity and mortality observed in these patients [17], [18], [19]. Particular focus was placed on ACEIs and ARBs, given emerging knowledge that SARS-CoV-2 accessed human pulmonary cells via ACE2. Hypotheses for ACEI/ARB interactions with COVID-19 have generally fallen along two axes: 1) ACEIs and/or ARBs might increase COVID-19 risk and severity by upregulating ACE2 in pulmonary tissue, thus providing greater opportunity for SARS-CoV-2 entry [20]; and, 2) ACEIs and/or ARBs may reduce severity of COVID-19 disease by shunting angiotensin II to angiotensin(1–7) via ACE2, resulting in anti-inflammatory effects that mitigate the cytokine storm associated with severe COVID-19 presentation. Early in the pandemic, concerns over the first hypothesis led to numerous suggestions, often amplified by high profile news outlets and social media, to discontinue ACEI/ARB therapy or switch to alternative antihypertensives. Nevertheless, in March 2020, most cardiovascular professional societies recommended continuation of these therapies unless further evidence emerged supporting adverse impacts on the clinical course of COVID-19 [21]. Since that time, many observational studies, both cohort and case control, have been reported, mostly from Chinese, European, and U.S. populations, associating ACEI/ARB vs. non-ACEI/ARB exposure with mortality and other severe outcomes. The vast majority of these studies have been summarized in recent meta-analyses, suggesting no effect of ACEI/ARBs on COVID-19 outcomes [22], or even a protective effect on some outcomes, including mortality [23], [24]. Many of the studies included in these meta-analyses have been small (hundreds of patients), employed biased study designs (e.g., introducing immortal time bias, or not including active comparators), or were inclusive only of early stages of the pandemic, often in places in which health systems were overwhelmed, introducing possible data validity issues [9], [20]. Nevertheless, the bulk of the evidence suggests that, at minimum, ACEI or ARB exposure is not associated with adverse outcomes among COVID-19-infected individuals, findings consistent with those observed here in a large, diverse population studied over most of 2020.

Given the large sample size, we were also able to directly compare ACEI- with ARB-exposed individuals to assess differential associations. Specifically, we observed ACEI-exposure associated with a 16% to 61% greater risk of all-cause hospitalization in the adjusted analysis, and no significant difference in risk of all-cause mortality in either the inpatient or outpatient cohorts. Results were similar in sensitivity analyses using a PS-matching approach. These findings generally accord with a prior analysis of patients in the Veterans Affairs (VA) system observed that ACEIs, as compared with ARBs, were associated with a 3%–14% greater risk of all-cause hospitalization or death among patients with COVID-19 diagnosed in the outpatient setting (adjusted HR, 0.92; 95% CI, 0.87, 0.98) [10]. That finding was primarily driven by greater risk of hospitalization among ACEI-exposed, as in our study. Although the design of the present study and the prior VA study preclude definitive causal conclusions, the replication of this finding suggests there may be differential effects of these drug classes on COVID-19 severity that require confirmation in randomized clinical trials. ARBs may interfere with the binding of the spike protein on SARS-CoV-2 and ACE2 and a recent small clinical trial among patients admitted to the ICU for COVID-19, found that telmisartan significantly reduced both time-to-discharge and mortality compared with standard care [25]. On the other hand, the significantly higher risk of UTI (one of two negative controls) in the ACEI- versus ARB-exposed groups may indicate residual confounding is responsible for at least part of the association observed between ACEI exposure and higher risk of hospitalization.

Finally, we performed stratified analyses for several pre-specified demographic and clinical criteria. We observed no evidence of effect modification by age, sex, race/ethnicity, baseline blood pressure, or baseline BMI. Although these analyses revealed a significant protective effect of ACEI/ARB exposure on all-cause hospitalization or all-cause death for the “other” race/ethnicity group (over half of whom were Asian Americans), the group was small (<5% of the outpatient cohort) and the interaction *p*-value was not significant (*p* = 0.12); thus, it seems plausible that this represents a chance finding. Taken together, these stratified analyses should provide some degree of certainty regarding the safety of continuing ACEI/ARB therapy in hypertensive individuals, regardless of their race/ethnicity, other demographic background, or their level of blood pressure control.

Our study has several strengths. First, our population was diverse, including more than 40% non-white individuals from 17 health care systems representing academic and community healthcare centers spanning rural and urban areas across many states. Furthermore, we employed propensity scores and IPTW-weighted analyses to approximate a randomized comparison and adjust for many potential confounders. Finally, we performed several sensitivity analyses to test the robustness of our results and included negative control outcomes to assess for potential confounding. Nevertheless, our analysis has important limitations. First, we used a prevalent-user design, similar to prior observational studies in this area. New-user designs are generally preferred in comparative drug effectiveness and safety studies [26]; however, such designs are challenging to implement in situations like the COVID-19 pandemic owing to insufficient numbers of new ACEI and ARB users with COVID-19 diagnoses over a short time-frame. Moreover, given the early coverage of concerns regarding ACEI/ARB use, new ACEI/ARB users, particularly early during the pandemic, may have represented a population perceived to be at lower risk by providers, which may have biased such an approach. Secondly, we employed prescribing data and dispensing data to identify antihypertensive exposure, including ACEI/ARB exposure. Although dispensing data are generally considered valid proxies for true exposure measurement, prescribing data may have greater measurement error due to non-persistence (i.e., never filling the original prescription) or non-adherence. Thirdly, we included individuals with compelling indications for ACEI/ARB therapy in the primary analysis, potentially introducing confounding by indication. However, remarkably similar results were observed in the outpatient cohort when we excluded individuals with compelling indications, suggesting that confounding by indication is unlikely to have demonstrably altered our main findings. Very few patients entered the inpatient cohort without compelling indications, and we cannot be certain whether confounding by indication may have played a role in the protective effect observed for ACEI/ARB exposure on mortality. However, presumably, any such confounding would have had an opposite effect (i.e., ACEI/ARB exposure appearing to have higher mortality risk) because most compelling indications for ACEI/ARB therapy are associated with mortality themselves, with more severe COVID-19 disease, or both. Fourth, although we included a large number of potential confounders in the PS model and performed analyses with negative control outcomes, we cannot exclude the possibility of residual confounding, particularly in the outpatient cohort where we compared ACEI vs. ARB exposure, as discussed previously. Relatedly, some variables used in the PS model had significant missingness, but in most cases, the overall proportion missing was strongly influenced by a small number of sites that did not provide any data for the specific variable. For example, several sites did not provide BP measurements, whereas for all others, BP data were available for ≥90% of patients. We used multiple imputation to address missingness, but it is possible that such an approach introduced additional uncertainty into our results. Fifth, the present study was performed using EHR data in non-vertically integrated health systems and patient care received outside of these health systems was not captured. By design, we excluded individuals who were not routine users of the respective healthcare system in which they were diagnosed, but we had no way of ensuring complete capture of relevant data. Finally, our results, particularly regarding hospitalization as an outcome, may need to be interpreted with some caution given the varying factors influencing decisions to hospitalize patients at certain times in certain locations during this pandemic.

In conclusion, in this real-world analysis of individuals with hypertension and COVID-19, we found no significant association with prior ACEI/ARB exposure, versus non-ACEI/ARB antihypertensive exposure, on all-cause hospitalization or death among individuals diagnosed in the outpatient setting, but a possible protective effect on mortality among inpatients. These findings are generally consistent with prior observational studies and clinical trials, suggesting no safety concerns for RAS inhibitors worsening the course of COVID-19 infections. Our findings of reduced hospitalizations among ARB-exposed (versus ACEI-exposed) has some support in the literature, but requires further study in larger, well-designed clinical trials before recommending switches from ACEI therapy.

## CRediT authorship contribution statement

**Steven M. Smith:** Conceptualization, Methodology, Formal analysis, Writing – original draft, Supervision, Funding acquisition. **Raj A. Desai:** Formal analysis, Visualization, Writing – review & editing. **Marta G. Walsh:** Data curation, Visualization, Writing – review & editing. **Ester Kim Nilles:** Resources, Data curation. **Katie Shaw:** Resources, Data curation. **Myra Smith:** Resources, Data curation. **Alanna M. Chamberlain:** Writing – review & editing. **Catherine G. Derington:** Methodology, Writing – review & editing. **Adam P. Bress:** Methodology, Writing – review & editing. **Cynthia H. Chuang:** Writing – review & editing. **Daniel E. Ford:** Writing – review & editing. **Bradley W. Taylor:** Writing – review & editing. **Sravani Chandaka:** Writing – review & editing. **Lav Parshottambhai Patel:** Writing – review & editing. **James McClay:** Writing – review & editing. **Elisa Priest:** Writing – review & editing. **Jyotsna Fuloria:** Writing – review & editing. **Kruti Doshi:** Writing – review & editing. **Faraz S. Ahmad:** Writing – review & editing. **Anthony J. Viera:** Writing – review & editing. **Madelaine Faulkner:** Project administration, Writing – review & editing. **Emily C. O'Brien:** Methodology, Writing – review & editing. **Mark J. Pletcher:** Methodology, Project administration, Funding acquisition, Resources, Writing – review & editing. **Rhonda M. Cooper-DeHoff:** Conceptualization, Methodology, Project administration, Funding acquisition, Resources, Writing – review & editing.

## Declaration of competing interest

The authors declare that they have no known competing financial interests or personal relationships that could have appeared to influence the work reported in this paper.

## Appendix A Supplementary data

Supplementary material Image 1
