# Supplementary material for: Angiotensin-converting enzyme inhibitors, angiotensin receptor blockers, and COVID-19-related outcomes: A patient-level analysis of the PCORnet blood pressure control lab
Source: Am Heart J Plus. 2022 Mar 2;13:100112. doi: 10.1016/j.ahjo.2022.100112 (PMC8889730; doi:10.1016/j.ahjo.2022.100112)
Supplement: Supplementary file 1 — Supplementary material [file mmc1.docx]

**SUPPLEMENT**

**Angiotensin-Converting Enzyme inhibitors, Angiotensin Receptor Blockers, and COVID-19-Related Outcomes: A Patient-level Analysis of the PCORnet Blood Pressure Control Lab**

Steven M. Smith, PharmD, MPH^1,2^, Raj Desai, MS^2^, Marta G. Walsh, MS^1^, Ester Kim Nilles, PhD^3^, Katie Shaw, MPH^4^, Myra Smith, MPH^4^, Alanna M. Chamberlain, PhD^5^, Catherine G. Derington, PharmD, MS^6^, Adam P. Bress, PharmD, MS^6^, Cynthia H. Chuang, MD, MS^7^, Daniel E. Ford, MD, MPH^8^, Bradley W. Taylor, MBA^9^, Sravani Chandaka, MS^10^, Lav Parshottambhai Patel, MS^10^, James McClay, MD, MS^11^, Elisa Priest, DrPH^12^, Jyotsna Fuloria, MD^13^, Kruti Doshi, MBA^14^, Faraz S. Ahmad, MD, MS^15^, Anthony J. Viera, MD, MPH^16^, Madelaine Faulkner, MPH^17^, Emily C. O’Brien, PhD^3^, Mark J. Pletcher, MD, MPH^17^, Rhonda M. Cooper-DeHoff, PharmD, MS^1^

^1^Department of Pharmacotherapy and Translational Research, College of Pharmacy, University of Florida, Gainesville, FL; ^2^Department of Pharmaceutical Outcomes and Policy, College of Pharmacy, University of Florida, Gainesville, FL; ^3^Duke Clinical Research Institute, Duke University, Durham, NC; ^4^Department of Health Outcomes and Biomedical Informatics, College of Medicine, University of Florida, Gainesville, FL; ^5^Departments of Quantitative Health Sciences and Cardiovascular Medicine, Mayo Clinic, Rochester, MN; ^6^Department of Population Health Sciences, School of Medicine, University of Utah, Salt Lake City, UT; ^7^Penn State University, Hershey, PA; ^8^Johns Hopkins University, Baltimore, MD; ^9^Medical College of Wisconsin, Milwaukee, WI; ^10^University of Kansas Medical Center, Kansas City, KS; ^11^University of Nebraska, Omaha, NE; ^12^Baylor Scott & White Health, Dallas, TX; ^13^School of Medicine, Louisiana State University, New Orleans, LA; ^14^Cook County Health, Chicago, IL; ^15^Departments of Medicine and Preventive Medicine, Feinberg School of Medicine, Northwestern University, Chicago, IL; ^16^Department of Family Medicine and Community Health, School of Medicine, Duke University, Durham, NC; ^17^Department of Epidemiology & Biostatistics, School of Medicine, University of California San Francisco, San Francisco, CA

**Corresponding Author**:

Dr. Steven M. Smith

University of Florida

PO Box 100486

Gainesville, FL

32610-0486

tel 352-273-5134

email [ssmith@cop.ufl.edu](mailto:ssmith@cop.ufl.edu)

**Supplemental Table S1. Participating Health Systems.**

| Pennsylvania State Medical Center |
| --- |
| Allina Health System |
| University of Kansas Medical Center |
| New York University |
| University of Florida |
| Mayo Clinic |
| Johns Hopkins University |
| Duke University |
| Medical College Wisconsin |
| University of Iowa |
| University of Nebraska |
| University of Utah |
| Baylor Scott & White North |
| University Medical Center New Orleans |
| Northwestern University |
| Cook County Health |
| Montefiore Medical Center |

**Supplemental Table S2. Current Procedural Technology (CPT) evaluation and management codes used to define outpatient and inpatient encounters.**

| **Setting** | **Evaluation and Management CPT codes** |
| --- | --- |
| Outpatient | 99201-99205, 99211-99215, 99495-99496, 99381-99387, 99391-99397 |
| Inpatient | 99217-99220, 99224-99226, 99221-99223, 99231-99233, 99238, 99239, 99251-99255, 99291, 99292, 99304-99310, 99315 |

**Supplemental Table S3. Antihypertensive drugs considered acceptable for inclusion in cohorts.**

| **Antihypertensive Class** | **Medication Name** |
| --- | --- |
| Angiotensin converting enzyme inhibitor | benazepril |
|  | captopril |
|  | enalapril |
|  | fosinopril |
|  | lisinopril |
|  | moexipril |
|  | perindopril |
|  | quinapril |
|  | ramipril |
|  | trandolapril |
| Angiotensin receptor blocker | azilsartan |
|  | candesartan |
|  | eprosartan |
|  | irbesartan |
|  | losartan |
|  | olmesartan |
|  | telmisartan |
|  | valsartan* |
| Alpha-blocker | doxazosin |
|  | prazosin |
|  | terazosin |
| Beta-blocker^†^ | acebutolol |
|  | atenolol |
|  | nadolol |
|  | oxprenolol |
|  | betaxolol |
|  | bisoprolol |
|  | carteolol |
|  | timolol |
|  | bucindolol |
|  | esmolol |
|  | labetalol |
|  | carvedilol |
|  | metoprolol |
|  | propranolol |
|  | nebivolol |
|  | penbutolol |
|  | pindolol |
|  | sotalol |
|  | metipranolol |
| Calcium channel blocker | amlodipine |
|  | felodipine |
|  | isradipine |
|  | nicardipine |
|  | nifedipine^‡^ |
|  | nisoldipine |
|  | diltiazem |
|  | verapamil |
| Centrally acting | clonidine |
|  | guanabenz |
|  | guanfacine |
|  | guanadrel |
|  | guanethidine |
|  | methyldopa |
|  | reserpine |
| Direct vasodilators | hydralazine |
|  | minoxidil |
| Direct renin inhibitor | aliskiren |
| Aldosterone receptor antagonist | spironolactone |
|  | eplerenone |
| Loop diuretic | bumetanide |
|  | ethacrynic acid |
|  | furosemide |
|  | torsemide |
| Potassium-sparing diuretic | amiloride |
|  | triamterene |
| Thiazide or Thiazide-like diuretic | bendroflumethiazide |
|  | chlorothiazide |
|  | chlorthalidone |
|  | hydrochlorothiazide |
|  | indapamide |
|  | metolazone |

*Excludes sacubitril/valsartan products.

^†^Excludes ophthalmologic products

^‡^Excludes nifedipine rectal ointment products.

**Supplemental Table S4. Variables Defining the Study Population.**

| **Variable** | **Definition** |
| --- | --- |
| Index date | Date of first occurrence of COVID-19 diagnosis on or after February 2020. Excludes any encounters missing DIAGNOSIS.ENCOUNTERID (i.e., diagnoses that cannot be linked back to a specific encounter) or an admission date. |
| COVID-19 Diagnosis | ICD-CM-10 diagnosis code U07.1 |
| Hypertension | Defined as any of the following (per DIAGNOSIS table):   1. ICD-CM-9 codes: ≥1 claim with a diagnosis code (any position) of 401.x, 403.0x, 403.1x, 403.9x. 2. ICD-CM-10 codes: ≥1 claim with a diagnosis code (any position) of I10, I12.0, I12.9. |

**Supplemental Table S5. Measurement Definitions for all Study Outcomes.**

| **Outcomes** | **Definition** |
| --- | --- |
| ***Primary*** |  |
| Primary composite outcome (outpatient cohort) | Composite of first occurrence of all-cause hospitalization or all-cause death. Time-to-event calculated as integer number of days from index date to date of death, first subsequent hospitalization, or censoring.^1^ |
| All-cause death (inpatient cohort) | Death, regardless of cause, ≥1 day following the index date. Patients dying on the index date were excluded. Time-to-event calculated as integer number of days from index date to death date or censoring date.^1^ |
|  |  |
| ***Secondary*** |  |
| All-cause hospitalization (outpatient cohort) | At least one encounter associated with ≥1 evaluation & management code denoted as hospitalization-related (per Supplemental Table S2).  Time-to-event calculated as integer number of days from index date to first all-cause hospitalization or censoring.^1^ By definition, patients with a hospitalization on the index date were included only in the inpatient cohort, regardless of whether an outpatient COVID-19 diagnosis existed on the same date. |
| All-cause death (outpatient cohort) | As above. |
| ICU Admission (inpatient cohort) | Occurrence of ≥1 of the following associated with the same hospitalization in which the index COVID-19 diagnosis occurred:  (a) Current Procedural Terminology E&M codes: 99291 or 99292  (b) OBSGEN table observation where OBSGEN_TYPE=”PC_COVID” and OBSGEN_CODE=”2000” and OBSGEN_SOURCE=”DR” and OBSGEN_RESULT_TEXT=”Y”.  Time-to-event calculated as integer number of days from index date to date of ICU admission or censoring.^2^ |
| Mechanical Ventilation  (inpatient cohort) | Occurrence of ≥1 of the following criteria associated with the same hospitalization in which the index COVID-19 diagnosis occurred:   1. ICD-10 codes: J95.850, Z99.1x, or T88.4x 2. Current Procedural Terminology: 31500 3. ICD-10 procedure codes: 5A19054, 5A0945Z, 5A1935Z, 5A1955Z, or 5A0935Z 4. OBSGEN table observation where OBSGEN_TYPE=”PC_COVID” and OBSGEN_CODE=”3000” and OBSGEN_SOURCE=”DR” and OBSGEN_RESULT_TEXT=”Y”   Time-to-event calculated as integer number of days from index date to first observation of mechanical ventilation criteria, or censoring.^2^ |
| Dialysis (inpatient cohort) | Occurrence of ≥1 of the following Dialysis associated with a patients first COVID diagnosis post-index date, inclusive of admit and discharge dates. Patients with an ICD-9 or ICD-10 code for end-stage renal disease (N18.6 or 585.5) at any time prior to the index date were excluded prior to calculation of this outcome. Patients meeting the ICU criteria outlined below prior to the index date were also excluded prior to calculation of this outcome. Occurrence of ≥1 of the following criteria associated with the same hospitalization in which the index COVID-19 diagnosis occurred, among patients without ICU admission during the same stay, and without prior evidence of end-stage renal disease (ICD-10 N18.6 or ICD-9 585.5) or dialysis at any time prior to the index date:   1. Current Procedural Terminology: 90935, 90937, 90945, 90947, 90999 2. ICD-10 procedure code: Z49.x   Time-to-event calculated as integer number of days from index date to first observation of dialysis criteria, or censoring.^2^ |
|  |  |
| ***Negative Control*** |  |
| Gastrointestinal bleeding  (both cohorts) | Occurrence of any of the following codes associated with a hospital (inpatient) encounter post-index date:   1. ICD-10 code (any position) K25.x, K26.x, K27.x, K28.x or K92.2. 2. CPT code 78278, 7424x, 7425x, 74260, 435xx, 436xx, 440xx, 44120, or 446xx   Time-to-event calculated as integer number of days from index date to first date meeting above criteria, or censoring. ^1^ |
| Urinary tract infection (both cohorts) | Presence of the ICD-10-CM codes N10, N30, N39, or A41.9 using all available claims post the index date.  Time-to-event calculated as integer number of days from index date to first date meeting above criteria, or censoring.^1^ |

^1^Censoring date was defined as the date of last encounter among all encounters within that health system, or December 9 2020 (date of the data query), whichever came first.

^2^Censoring date was defined as the date of discharge from the index hospitalization, or absent a discharge date, the last encounter among all encounters within that health system, or December 9 2020 (date of the data query), whichever came first.

**Supplemental Table S6. Definitions for study covariates.**

| **Variable** | **Definition** |
| --- | --- |
| Age | Age calculated on the index date based on date of birth available in DEMOGRAPHIC.BIRTH_DATE. |
| Sex | As per DEMOGRAPHIC.SEX |
| Race-ethnicity | As per DEMOGRAPHIC.RACE and DEMOGRAPHIC.HISPANIC |
| Current Smoking | Any of the following within one year prior to the index date (including the index date):   1. Most recent VITAL.SMOKING in 01, 02, 07, or 08 2. ICD-9 codes:    1. ≥1 hospitalization with a discharge diagnosis code (any position) of tobacco use of 305.1, 649.0x, 989.84, or V15.82) in any discharge position    2. ≥1 physician evaluation and management visit with a discharge diagnosis code (any position) of tobacco use of 305.1, 649.0x, 989.84, or V15.82) in any discharge position 3. ICD-10 codes:    1. ≥1 hospitalization with a discharge diagnosis code (any position) of tobacco use of F17.200, F17.201, F17.210, F17.211, F17.220, F17.221, F17.290, F17.291, or Z87.891) in any discharge position    2. ≥1 outpatient visit with a discharge diagnosis code (any position) of tobacco of F17.200, F17.201, F17.210, F17.211, F17.220, F17.221, F17.290, F17.291, or Z87.891) in any discharge position 4. ≥1 visit with an evaluation and management code and evidence of tobacco use, identified via CPT code (any position) of 99406, 99407, G0436, G0437, G9016, S9453, S4995, G9276, G9458, 1034F, 4004F, or 4001F 5. ≥1 pharmacy prescription or fill for nicotine or varenicline in the 365 days before the index date (including the index date). |
| Insurance type | As per ENCOUNTER.PAYER_TYPE_PRIMARY on SARS-CoV-2-positive diagnosis encounter (if specified). Categorized as Medicare, Medicaid, Other Government, Commercial Insurance and Managed Care, Self-pay or charity care, Other, or Unknown. |
| Height | As per VITAL.HT, height (in inches) on the index date or the date closest to the index date during the one-year pre-index period, else most recent height at any date prior to the index date. Retain values that are within 3 SD of the mean of all heights in the cohort. If only observations >3 SD of the mean are populated, and ≥2 such identical observations exist, use the value as patient height. Otherwise, code as missing. |
| Weight | As per VITAL.WT, weight (in lbs) on the index date or the date closest to the index date during the one-year pre-index period. Retain values that are within 3 SD of the mean of all weights in the cohort. If only observations >3 SD of the mean are populated, and ≥2 such observations exist that are within 5 lbs of each other, use the more recent of the two as patient weight. Otherwise, code as missing. |
| Body mass index | As per VITAL.ORIGINAL_BMI, Body mass index on or closest to the index date during the one-year pre-index period. For patients without a valid value for ORIGINAL_BMI, BMI can be derived from the most recent height and weight measurements (even if not same day) as weight (in kg) divided by height (in meters) squared. |
| Blood pressure | As per VITAL.SYSTOLIC and DIASTOLIC, Systolic and Diastolic BP values on or closest to the index date during the one-year pre-index period that correspond to an outpatient encounter. SBP and DBP values should be dropped if SBP < DBP, SBP is >300 mmHg or <50 mmHg, or DBP is >180 mmHg or <30 mmHg. |
| Total cholesterol | Total cholesterol value closest to the index date in the one-year pre-index period. Defined using LAB_RESULT_CM.LAB_LOINC codes 2093-3, 2565-0, or 50339-1. |
| HDL-C level | HDL-C value closest to the index date in the one-year pre-index period. Defined by LAB_RESULT_CM.LAB_LOINC codes 2085-9, 2086-7, or 49130-8. |
| LDL-C level | LDL-C value closest to the index date in the one-year pre-index period. Defined using LAB_RESULT_CM.LAB_LOINC codes 13457-7, 18262-6, 2089-1, 2090-9, 49132-4, or 55440-2. |
| Triglyceride level | Triglyceride value closest to the index date in the one-year pre-index period. Defined using LAB_RESULT_CM.LAB_LOINC codes 2571-8, 3043-7, or 3049-4. |
| Hemoglobin A1c | Glycated hemoglobin value closest to the index date in the one-year pre-index period. Defined using LAB_RESULT_CM.LAB_LOINC codes 4548-4, 17855-8, 4549-2, or 17856-6. Result number values restricted to range: (0,20]. |
| Serum creatinine | Serum creatinine value closest to the index date in the one-year pre-index period. Defined using LAB_RESULT_CM.LAB_LOINC codes 2160-0, 21232-4, or 38483-4. Excludes results where RESULT_UNIT=mg/(24.h). Results where the RESULT_UNIT=mmol/L are converted to mg/dL by RESULT_NUM/88.42. |
| Estimated glomerular filtration rate | Estimated glomerular filtration rate closest to the index date in the one-year pre-index period. Defined using LAB_RESULT_CM.LAB_LOINC codes 76633-7, 33914-3, 69405-9, 94677-2, 62238-1, 77147-7, 50384-7, 50210-4, 88293-6, 48643-1, 50044-7, 70969-1, 88294-4, or 48642-3. To account for data/reading errors, only values [0,250] are considered valid. Additional caveats apply:   1. Exclude any results where the RESULT_UNIT=mg/dL or the RESULT_MODIFIER in (GE, GT, LE, LT). 2. If patient is black, exclude LAB_LOINC = 48642-3 and 88294-4. 3. If patient is non-black, exclude LAB_LOINC = 48643-1 or 88293-6.   If patient is male, exclude LAB_LOINC = 50044-7. |
| Serum potassium | Serum potassium value closest to the index date in the one-year pre-index period. Defined using LAB_RESULT_CM.LAB_LOINC codes 6298-4, 2823-3, or 22760-3. |
| Diabetes | Any of the following using all available claims prior to the index date (including the index date):   1. ICD-9 codes:    1. ≥1 inpatient claim with a discharge diagnosis code (any position) of 250.xx, 357.2, 362.0x, or 366.41.    2. At least 2 outpatient claims with diagnosis code (any position) of 250.xx, 357.2, 362.0x, or 366.41, with the 2 claims occurring at least 7 days apart. 2. ICD-10 codes:    1. ≥1 inpatient claim with a discharge diagnosis code (any position) of E0836, E08.42, E09.36, E09.42, E10.10, E10.11, E10.29, E10.311, E10.319, E10.36, E10.39, E10.40, E10.42, E10.51, E10.618, E10.620, E10.621, E10.622, E10.628, E10.630, E10.638, E10.641, E10.649, E10.65, E10.69, E10.8, E10.9, E11.00, E11.01, E11.29, E11.311, E11.319, E11.329, E11.339, E11.349, E11.359, E11.36, E11.39, E11.40, E11.42, E11.51, E11.618, E11.620, E11.621, E11.622, E11.628, E11.630, E11.638, E11.641, E11.649, E11.65, E11.69, E11.8, E11.9, E13.10, E13.36, E13.42.    2. At least 2 outpatient claims with diagnosis code (any position) of E0836, E08.42, E09.36, E09.42, E10.10, E10.11, E10.29, E10.311, E10.319, E10.36, E10.39, E10.40, E10.42, E10.51, E10.618, E10.620, E10.621, E10.622, E10.628, E10.630, E10.638, E10.641, E10.649, E10.65, E10.69, E10.8, E10.9, E11.00, E11.01, E11.29, E11.311, E11.319, E11.329, E11.339, E11.349, E11.359, E11.36, E11.39, E11.40, E11.42, E11.51, E11.618, E11.620, E11.621, E11.622, E11.628, E11.630, E11.638, E11.641, E11.649, E11.65, E11.69, E11.8, E11.9, E13.10, E13.36, E13.42, with the 2 claims occurring at least 7 days apart. 3. ≥1 prescription or fill for an oral or injectable antidiabetic drug in the one-year pre-index period. 4. Positive indication of T2DM via the PCORnet-validated T2DM phenotype |
| Chronic kidney disease | Any of the following using all available diagnoses prior to the index date (including the index date):   1. ICD-9 codes:    1. ≥1 inpatient claim with a discharge diagnosis code (any position) of (585.5, 585.1, 582.9, 582.81, 582.2, 585.9, 581, 404.9, 403.9, 403.1, 249.41, 637.32, 637.31, 580, 442.1, 404.1, 250.43, 249.4, 581.3, 404.12, 404, 403, 250.42, 582.89, 582.4, 582, 581.1, 404.02, 403.11, 250.4, 639.3, 585.2, 585, 580.81, 586, 581.9, 581.81, 403.91, 585.6, 585.3, 582.1, 580.8, 403.01, 582.8, 580.89, 404.93, 250.41, 637.3, 404.92, 585.4, 581.89, 581.8, 580.9, 404.13, 581.2, 404.91, 404.11, 404.03, 404.01).    2. At least 2 outpatient claims with diagnosis code (any position) of (585.5, 585.1, 582.9, 582.81, 582.2, 585.9, 581, 404.9, 403.9, 403.1, 249.41, 637.32, 637.31, 580, 442.1, 404.1, 250.43, 249.4, 581.3, 404.12, 404, 403, 250.42, 582.89, 582.4, 582, 581.1, 404.02, 403.11, 250.4, 639.3, 585.2, 585, 580.81, 586, 581.9, 581.81, 403.91, 585.6, 585.3, 582.1, 580.8, 403.01, 582.8, 580.89, 404.93, 250.41, 637.3, 404.92, 585.4, 581.89, 581.8, 580.9, 404.13, 581.2, 404.91, 404.11, 404.03, 404.01)., with the 2 claims occurring at least 7 days apart. 2. ICD-10 codes:    1. ≥1 inpatient claim with a discharge diagnosis code (any position) of E11.29, N26.2, E10.29, I13.11, E11.22, E11.21, O10.311, O10.213, O10.33, O10.312, E09.29, O10.211, M32.14, E13.22, E09.22, O10.32, O10.313, E13.29, E10.21, E09.21, E08.22, Q87.81, O10.212, N18.6, E10.22, I13.10, E13.21, O10.319, O10.23, O10.22, O10.219, E08.21, P96.0, N99.0, N19, N18.9, N18.5, N18.4, N18.3, N18.2, N18.1, N07.7, N07.5, N07.4, N07.3, N07.2, N05.9, N05.8, N05.7, N05.6, N05.5, N05.4, N05.3, N05.2, N05.1, N05.0, N04.9, N04.8, N04.7, N04.6, N04.5, N04.4, N04.3, N04.2, N04.1, N04.0, N03.9, N03.8, N03.7, N03.6, N03.5, N03.4, N03.3, N03.2, N03.1, N03.0, N02.7, N02.6, N02.4, N00.7, I72.2, I13.2, I13.0, I12.9, I12.0, O10.31, O10.3, O10.21, O10.2, N18, N05, N04, N03, I13.1, I13, I12, E13.2, E11.2, E10.2, E09.2) .    2. ≥1 outpatient claim with a diagnosis code (any position) of E11.29, N26.2, E10.29, I13.11, E11.22, E11.21, O10.311, O10.213, O10.33, O10.312, E09.29, O10.211, M32.14, E13.22, E09.22, O10.32, O10.313, E13.29, E10.21, E09.21, E08.22, Q87.81, O10.212, N18.6, E10.22, I13.10, E13.21, O10.319, O10.23, O10.22, O10.219, E08.21, P96.0, N99.0, N19, N18.9, N18.5, N18.4, N18.3, N18.2, N18.1, N07.7, N07.5, N07.4, N07.3, N07.2, N05.9, N05.8, N05.7, N05.6, N05.5, N05.4, N05.3, N05.2, N05.1, N05.0, N04.9, N04.8, N04.7, N04.6, N04.5, N04.4, N04.3, N04.2, N04.1, N04.0, N03.9, N03.8, N03.7, N03.6, N03.5, N03.4, N03.3, N03.2, N03.1, N03.0, N02.7, N02.6, N02.4, N00.7, I72.2, I13.2, I13.0, I12.9, I12.0, O10.31, O10.3, O10.21, O10.2, N18, N05, N04, N03, I13.1, I13, I12, E13.2, E11.2, E10.2, E09.2), with the 2 claims occurring at least 7 days apart. 3. Estimated glomerular filtration rate of <60 mL/min/1.73 m^2^.The eGFR value used is the most recent within the one-year pre-index period. To account for data/reading errors, only eGFR values between 0 and 59.9 will be considered. Defined using LOINC GROUP code: LG50986-5 - LONIC code in (76633-7, 33914-3, 69405-9, 94677-2, 62238-1, 77147-7, 50384-7, 50210-4, 88293-6, 48643-1, 50044-7, 70969-1, 88294-4, 48642-3). Additional caveats include: 4. Exclude any results where the result_unit=mg/dL or the result_modifier in (GE, GT, LE, LT). 5. If patient is black, exclude LAB_LOINC = 48642-3 and 88294-4. 6. If patient is non-black, exclude LAB_LOINC = 48643-1 or 88293-6. 7. If patient is male, exclude LAB_LOINC = 50044-7. 8. Positive indication of CKD via the eMERGE-validated phenotype |
| Heart failure with reduced ejection fraction | Any one of the following using all available claims before the index date:   1. ICD-9 codes:    1. ≥1 inpatient claim with discharge diagnosis code (any position) of 428.0x, 428.1x, 428.2x, or 428.4x.    2. ≥2 outpatient claims on separate calendar days with diagnosis code (any position) of 428.0x, 428.1x, 428.2x, or 428.4x. 2. ICD-10 codes:    1. ≥1 inpatient claim with discharge diagnosis code (any position) of I50.1, I50.2x, I50.4x, or I50.9.    2. ≥2 outpatient claims on separate calendar days with diagnosis code (any position) of I50.1, I50.2x, I50.4x, or I50.9. 3. At least one prescription for sacubitril/valsartan in the 104 days prior to the index date. |
| History of CHD | Any of the following using all available claims prior to the index date (including the index date):   1. ICD-9 codes:    1. ≥1 inpatient encounter with a discharge diagnosis code (any position) of 410.xx-414.xx, V45.81, or V45.82.    2. At least 2 outpatient encounter with diagnosis code (any position) of 410.xx-414.xx, V45.81, or V45.82. 2. ICD-10 codes:    1. ≥1 inpatient encounter with a discharge diagnosis code (any position) of I20.0, I21.xx, I22.xx, I24.0, I24.8, I24.9, I25.10, I25.110, I25.700, I25.710, I25.720, I25.730, I25.750, I25.760, I25.790, I25.810, I25.811, I25.812, I25.3, I25.41, I25.42, Z95.1, or Z9861.    2. At least 2 outpatient encounters with diagnosis codes of codes I20.0, I21.xx, I22.xx, I24.0, I24.8, I24.9, I25.10, I25.110, I25.700, I25.710, I25.720, I25.730, I25.750, I25.760, I25.790, I25.810, I25.811, I25.812, I25.3, I25.41, I25.42, Z95.1, or Z98.61.   Patients who met the definition of a prior coronary revascularization, as defined below, are also considered to have a history of CHD. |
| Prior coronary revascularization | Defined by ≥1 inpatient or outpatient procedure with a CPT code for coronary revascularization (33510-33519, 33521-33523, 33530, 33533-33536, 92920, 92921, 92924, 92925, 92928, 92929, 92933, 92934, 92937, 92938, 92941, 92943, 92944, 92980, 92981, 92982, 92984, or 92996), an ICD-9 procedure code (any position) of 00.66, 36.0, 36.01-36.19, or 36.2, or an ICD-10 procedure code starting with any of the following: 0210, 0211, 0212, 0213, 0270, 0271, 0272, 0273, 02C0, 02C1, 02C2, 02C3, or 3E07 using all available claims prior to the index date (including the index date). In addition to having 1 inpatient or outpatient procedure, patients are required to meet ≥1of the following criteria:   1. Have no inpatient claims with a discharge diagnosis code for acute myocardial infarction (ICD-9 codes 410.x0 or 410.x1 or ICD-10 codes I21.xx or I22.xx) within 60 days prior to the procedure. 2. Have primary discharge diagnosis code for non-elective CHD-related hospitalization prior to the index date (including the index date): 3. Arrhythmia: ICD-9 diagnosis code of 427.xx [except 427.5] or ICD-10 diagnosis code of I47.1, I47.2, I47.9, I48.91, I48.92, I49.01, I49.02, I49.1, I49.3, I49.40, I49.49, I49.5, I49.8, I49.9, R00.1. 4. Cardiac arrest: ICD-9 diagnosis code of 427.5, or ICD-10 diagnosis code of I46.9. 5. Heart failure: ICD-9 diagnosis code of 402.01, 402.11, 402.91, 404.01, 404.03, 404.11, 404.13, 404.91, 404.93, or 428.x, or ICD-10 diagnosis code of I11.0, I13.0, I13.2, I50.1, I50.20, I50.21, I50.22, I50.23, I50.30, I50.31, I50.32, I50.33, I50.40, I50.41, I50.42, I50.43, or I50.9. 6. Unstable angina: ICD-9 diagnosis code of 411.xx or ICD-10 diagnosis code of I20.0, I24.0, I24.1, I24.8. |
| History of Stroke | Any of the following using all available claims prior to the index date (including the index date):   1. ICD-9 codes:    1. ≥1 inpatient claim with a discharge diagnosis code in the primary or secondary position of 433.x1 or 434.x1.    2. ≥1 outpatient claim with a diagnosis code (any position) of 433.x1 or 434.x1. 2. ICD-10 codes:    1. ≥1 inpatient discharge diagnosis code in the primary or secondary position of I63.xx.    2. ≥1 outpatient claim with diagnosis code (any position) of I63.xx.    3. ≥1 inpatient ICD-10 procedure code of 03CH0ZZ, 03CH4ZZ, 03CJ0ZZ, 03CJ4ZZ, 03CK0ZZ, 03CK4ZZ, 03CL0ZZ, 03CL4ZZ, 03CM0ZZ, 03CM4ZZ, 03CN0ZZ, 03CN4ZZ, 03RH07Z, 03RH0JZ, 03RH0KZ, 03RH47Z, 03RH4JZ, 03RH4KZ, 03RJ07Z, 03RJ0JZ, 03RJ0KZ, 03RJ47Z, 03RJ4JZ, 03RJ4KZ,03RK07Z, 03RK0JZ, 03RK0KZ, 03RK47Z, 03RK4JZ, 03RK4KZ, 03RL07Z, 03RL0JZ, 03RL0KZ, 03RL47Z, 03RL4JZ, 03RL4KZ, 03RM07Z, 03RM0JZ, 03RM0KZ, 03RM47Z, 03RM4JZ, 03RM4KZ, 03RN07Z, 03RN0JZ, 03RN0KZ, 03RN47Z, 03RN4JZ, or 03RN4KZ. 3. CPT codes: ≥1 inpatient or outpatient claim with a CPT code for carotid revascularization of 35301, 35390, 37215, 37216, 0005T, 0075T, or 0076. |
| History of PAD | Any of the following using all available claims prior to the index date (including the index date):   1. ICD-9 codes:    1. ≥1 inpatient claim with a discharge diagnosis code (any position) of 440.20-440.24, 440.31, 444.2, 443.9, or 444.81.    2. ≥2 physician evaluation and management or outpatient claims with diagnosis code (any position) of 440.20-440.24, 440.31, 444.2, 443.9, or 444.81 on separate days. 2. ICD-10 codes:    1. ≥1 inpatient claim with a discharge diagnosis code (any position) of I70.209, I70.219, I70.229, I70.25, I70.269, I70.499, I73.9.    2. ≥2 physician evaluation and management or outpatient claims with a diagnosis code (any position) of I70.209, I70.219, I70.229, I70.25, I70.269, I70.499, I73.9 on separate days. 3. CPT codes: ≥1 inpatient or outpatient claim with a CPT code of 37205 or 75962. |
| History of ASCVD | Defined by a history of CHD, cerebrovascular disease, or peripheral artery disease, as defined above. |
| End-stage renal disease | Any of the following using all available claims prior to the index date (including the index date):   1. ICD-9 codes:    1. ≥1 inpatient claim with a discharge diagnosis code (any position) of 585.5.    2. ≥2 physician evaluation and management or outpatient claims with a diagnosis code (any position) of 585.5. 2. ICD-10 codes:    1. ≥1 inpatient claim with a discharge diagnosis code (any position) of N18.6.    2. ≥2 physician evaluation and management or outpatient claims with a diagnosis code (any position) of N18.6. |
| History of kidney transplant | Any of the following using all available claims prior to the index date (including the index date):   1. ICD-9 codes:    1. ≥1 inpatient claim with a discharge diagnosis code (any position) of V42.0.    2. ≥2 physician evaluation and management or outpatient claims with a diagnosis code (any position) of V42.0. 2. ICD-10 codes:    1. ≥1 inpatient claim with a discharge diagnosis code (any position) of Z94.0.    2. ≥2 physician evaluation and management or outpatient claims with a diagnosis code (any position) of Z94.0. |
| Atrial fibrillation | Any of the following using all available claims prior to the index date (including the index date):   1. ICD-9 codes:    1. ≥1 inpatient claim with a discharge diagnosis code (any position) of 427.31.    2. ≥2 physician evaluation and management or outpatient claims with a diagnosis code (any position) of 427.31. 2. ICD-10 codes:    1. ≥1 inpatient claim with a discharge diagnosis code (any position) of I48.0, I48.2, I48.91.    2. ≥2 physician evaluation and management or outpatient claims with a diagnosis code (any position) of I48.0, I48.2, I48.91. |
| Chronic obstructive pulmonary disease | Any of the following using all available claims prior to the index date (including the index date):   1. ICD-9 codes:    1. ≥1 inpatient claim with a discharge diagnosis code (any position) of 491.x or 492.x or 496.x.    2. ≥2 physician evaluation and management or outpatient claims with a diagnosis code (any position) of 491.x or 492.x or 496.x. 2. ICD-10 codes:    1. ≥1 inpatient claim with a discharge diagnosis code (any position) of J41.x, J42.x, J43.x, or J44.x.    2. ≥2 physician evaluation and management or outpatient claims with a diagnosis code (any position) of J41.x, J42.x, J43.x, or J44.x. |
| Asthma | Any of the following using all available claims prior to the index date (including the index date):   1. ICD-9 codes:    1. ≥1 inpatient claim with a discharge diagnosis code (any position) of 493.x.    2. ≥2 physician evaluation and management or outpatient claims with a diagnosis code (any position) of 493.x. 2. ICD-10 codes:    1. ≥1 inpatient claim with a discharge diagnosis code (any position) of J45.x.    2. ≥2 physician evaluation and management or outpatient claims with a diagnosis code (any position) of J45.x. |
| History of depression | Any of the following using all available claims prior to the index date (including the index date):   1. ICD-9 codes:    1. ≥1 inpatient claim with a discharge diagnosis code (any position) of 296.2, 296.3, 296.5, 300.4, 309.x, or 311.    2. ≥2 physician evaluation and management or outpatient claims with a diagnosis code (any position) of 296.2, 296.3, 296.5, 300.4, 309.x, or 311. 2. ICD-10 codes:    1. ≥1 inpatient claim with a discharge diagnosis code (any position) of F20.4, F31.3-F31.5, F32.x, F33.x, F34.1, F41.2, or F43.2.    2. ≥2 physician evaluation and management or outpatient claims with a diagnosis code (any position) of F20.4, F31.3-F31.5, F32.x, F33.x, F34.1, F41.2, or F43.2. |
| Charlson Comorbidity Score | Continuous variable to represent chronic disease burden. Calculated according to Elixhauser method using publically available MINI-SENTINEL software. |
| Current statin use | Defined as one or more prescriptions (PRESCRIBING) or fills (DISPENSING) for a statin medication in the 90 days prior to the index date. |
| Current aspirin use | Defined as one or more prescriptions (PRESCRIBING) or fills (DISPENSING) for aspirin in the 90 days prior to the index date. |
| Current outpatient anticoagulant use | Defined as one or more prescriptions (PRESCRIBING) or fills (DISPENSING) for anticoagulants in the 90 days prior to the index date. |

**Supplemental Table S7. Baseline characteristics of ACEI- and ARB-exposed individuals in the outpatient cohort.**

| **Baseline Characteristic** | **Overall Cohort**  (n=7,450) | **ACEI-Exposed**  (n=3,838) | **ARB-Exposed**  (n=3,612) |
| --- | --- | --- | --- |
| ***Demographics*** |  |  |  |
| Age, years | 61.6 ± 12.3 | 60.4 ± 12.6 | 62.9 ± 11.8 |
| <45 | 663 (9%) | 422 (11%) | 241 (7%) |
| 45-64 | 3,621 (49%) | 1,927 (50%) | 1,694 (47%) |
| ≥65 | 3,166 (42%) | 1,489 (39%) | 1,677 (46%) |
| Sex |  |  |  |
| Female | 3,964 (53%) | 1,924 (50%) | 2,040 (56%) |
| Male | 3,485 (47%) | 1,914 (50%) | 1,571 (43%) |
| Unknown | 1 (0%) | 0 (0%) | 1 (0%) |
| Race, self-reported |  |  |  |
| American Indian or Alaska Native | 48 (1%) | 31 (1%) | 17 (0%) |
| Asian | 208 (3%) | 84 (2%) | 124 (3%) |
| Black or African American | 1,828 (25%) | 939 (24%) | 889 (25%) |
| Native Hawaiian or Other Pacific Islander | 29 (0%) | 15 (0%) | 14 (0%) |
| White | 4,263 (57%) | 2,199 (57%) | 2,064 (57%) |
| Multiple races | 53 (1%) | 20 (1%) | 33 (1%) |
| Unknown | 1,021 (14%) | 550 (14%) | 471 (13%) |
| Ethnicity |  |  |  |
| Non-Hispanic | 5,842 (78%) | 2,977 (78%) | 2,865 (79%) |
| Hispanic | 1,219 (16%) | 709 (18%) | 510 (14%) |
| Unknown |  |  |  |
| Height, inches | 66.4 ± 4.3 | 66.7 ± 4.4 | 66.2 ± 4.2 |
| Missing data | 1,243 (17%) | 708 (18%) | 535 (15%) |
| Weight, pounds | 192.3 ± 1.9 | 192.4 ± 1.8 | 192.2 ± 1.9 |
| Missing data | 6,574 (88%) | 3,370 (88%) | 3,204 (89%) |
| Body mass index, kg/m^2^ | 33.0 ± 8.0 | 33.0 ± 8.7 | 33.0 ± 7.3 |
| Missing data | 3,249 (44%) | 1,765 (46%) | 1,484 (41%) |
|  |  |  |  |
| ***Vitals & Labs*** |  |  |  |
| Blood pressure, mm Hg |  |  |  |
| Systolic | 133 ± 18 | 133 ± 18 | 134 ± 18 |
| Diastolic | 78 ± 11 | 78 ± 11 | 78 ± 11 |
| Missing BP data | 2,458 (33%) | 1,008 (26%) | 1,450 (40%) |
| Total cholesterol, mg/dL | 170 ± 46 | 171 ± 47 | 169 ± 44 |
| Missing data | 2,650 (36%) | 1,463 (38%) | 1,187 (33%) |
| HDL-C, mg/dL | 50 ± 15 | 49 ± 15 | 51 ± 15 |
| Missing data | 3,127 (42%) | 1,723 (45%) | 1,404 (39%) |
| LDL-C, mg/dL | 95 ± 36 | 96 ± 37 | 93 ± 35 |
| Missing data | 2,738 (37%) | 1,523 (40%) | 1,215 (34%) |
| Triglyceride, mg/dL | 144 ± 103 | 146 ± 107 | 142 ± 100 |
| Missing data | 2,812 (38%) | 1,572 (41%) | 1,240 (34%) |
| Hemoglobin A1c, % | 6.85 ± 1.70 | 7.04 ± 1.81 | 6.67 ± 1.55 |
| Missing data | 3,113 (42%) | 1,688 (44%) | 1,425 (39%) |
| Serum creatinine, mg/dL | 1.00 ± 0.44 | 0.99 ± 0.39 | 1.01 ± 0.49 |
| Missing data | 1,259 (17%) | 708 (18%) | 551 (15%) |
| Estimated GFR, mL/min/1.73m^2^ | 73.63 ± 24.87 | 74.05 ± 25.07 | 73.23 ± 24.68 |
| Missing data | 3,758 (50%) | 2,039 (53%) | 1,719 (48%) |
| Serum potassium, mg/dL | 4.24 ± 0.47 | 4.26 ± 0.48 | 4.21 ± 0.47 |
| Missing data | 1,526 (20%) | 840 (22%) | 686 (19%) |
|  |  |  |  |
| ***Comorbidities*** |  |  |  |
| Current Smoking | 1,069 (14%) | 624 (16%) | 445 (12%) |
| Diabetes | 3,455 (46%) | 1,853 (48%) | 1,602 (44%) |
| Chronic kidney disease | 1,902 (26%) | 943 (25%) | 959 (27%) |
| End-stage renal disease | 3 (0%) | 2 (0%) | 1 (0%) |
| History of kidney transplant | 5 (0%) | 1 (0%) | 4 (0%) |
| Heart failure with reduced EF | 371 (5%) | 223 (6%) | 148 (4%) |
| History of CHD | 1,138 (15%) | 559 (15%) | 579 (16%) |
| Prior coronary revascularization | 87 (1%) | 52 (1%) | 35 (1%) |
| History of Stroke | 256 (3%) | 110 (3%) | 146 (4%) |
| History of PAD | 204 (3%) | 113 (3%) | 91 (3%) |
| History of ASCVD | 1,404 (19%) | 685 (18%) | 719 (20%) |
| Atrial fibrillation | 393 (5%) | 190 (5%) | 203 (6%) |
| COPD | 477 (6%) | 220 (6%) | 257 (7%) |
| Asthma | 875 (12%) | 395 (10%) | 480 (13%) |
| History of depression | 1,154 (15%) | 630 (16%) | 524 (15%) |
| Charlson Comorbidity Score | 2.29 ± 3.11 | 2.28 ± 3.12 | 2.29 ± 3.10 |
|  |  |  |  |
| ***Medication Use*** |  |  |  |
| Statin | 2,227 (30%) | 1,163 (30%) | 1,064 (29%) |
| Aspirin | 726 (10%) | 376 (10%) | 350 (10%) |
| Anticoagulants | 585 (8%) | 316 (8%) | 269 (7%) |
| *Antihypertensives* |  |  |  |
| ACE inhibitor | 3,838 (52%) | 3,838 (100%) | 0 (0%) |
| ARB | 3,612 (48%) | 0 (0%) | 3,612 (100%) |
| Direct renin inhibitor | 1 (0%) | 0 (0%) | 1 (0%) |
| Aldosterone receptor antagonist | 327 (4%) | 142 (4%) | 185 (5%) |
| Dihydropyridine CCB | 2,318 (31%) | 1,113 (29%) | 1,205 (33%) |
| Non-dihydropyridine CCB | 276 (4%) | 122 (3%) | 154 (4%) |
| Thiazide diuretic | 2,931 (39%) | 1,313 (34%) | 1,618 (45%) |
| Loop diuretic | 866 (12%) | 444 (12%) | 422 (12%) |
| Potassium-sparing diuretic | 146 (2%) | 61 (2%) | 85 (2%) |
| β-blocker | 2,634 (35%) | 1,297 (34%) | 1,337 (37%) |
| α_1_ blocker | 146 (2%) | 72 (2%) | 74 (2%) |
| α­_2_ agonist | 142 (2%) | 58 (2%) | 84 (2%) |
| Direct vasodilator | 320 (4%) | 154 (4%) | 166 (5%) |
|  |  |  |  |
| ***Insurance Type*** |  |  |  |
| Medicaid | 304 (4%) | 199 (5%) | 105 (3%) |
| Medicare | 1,070 (14%) | 563 (15%) | 507 (14%) |
| Other Government | 134 (2%) | 73 (2%) | 61 (2%) |
| Commercial Insurance or Managed Care | 1,289 (17%) | 733 (19%) | 556 (15%) |
| Self-pay or charity care | 113 (2%) | 80 (2%) | 33 (1%) |
| Other | 43 (1%) | 25 (1%) | 18 (0%) |
| Unknown | 616 (8%) | 340 (9%) | 276 (8%) |
| Missing data | 3,881 (52%) | 1,825 (48%) | 2,056 (57%) |

ACEI, angiotensin-converting enzyme inhibitor; ARB, angiotensin receptor blocker; ASCVD, atherosclerotic cardiovascular disease; CCB, calcium channel blocker; CHD, coronary heart disease; COPD, chronic obstructive pulmonary disease; EF, ejection fraction; GFR, glomerular filtration rate; HDL-C, high-density lipoprotein cholesterol; LDL-C, low-density lipoprotein cholesterol; PAD, peripheral arterial disease.

**Supplemental Table S8. Baseline characteristics of ACEI- and ARB-exposed individuals in the Inpatient Cohort.**

| **Baseline Characteristic** | **Overall Cohort**  (n=1,407) | **ACEI- Exposed**  (n=790) | **ARB-Exposed**  (n=617) |
| --- | --- | --- | --- |
| ***Demographics*** |  |  |  |
| Age, years | 66.2 ± 12.1 | 64.8 ± 12.4 | 68.1 ± 11.4 |
| <45 | 71 (5%) | 54 (7%) | 17 (3%) |
| 45-64 | 507 (36%) | 298 (38%) | 209 (34%) |
| ≥65 | 829 (59%) | 438 (55%) | 391 (63%) |
| Sex |  |  |  |
| Female | 701 (50%) | 362 (46%) | 339 (55%) |
| Male | 706 (50%) | 428 (54%) | 278 (45%) |
| Race, self-reported |  |  |  |
| American Indian or Alaska Native | 19 (1%) | 10 (1%) | 9 (1%) |
| Asian | 31 (2%) | 13 (2%) | 18 (3%) |
| Black or African American | 427 (30%) | 235 (30%) | 192 (31%) |
| Native Hawaiian or Other Pacific Islander | 5 (0%) | 3 (0%) | 2 (0%) |
| White | 758 (54%) | 430 (54%) | 328 (53%) |
| Multiple races | 11 (1%) | 4 (1%) | 7 (1%) |
| Unknown | 156 (11%) | 95 (12%) | 61 (10%) |
| Ethnicity |  |  |  |
| Non-Hispanic | 1,203 (86%) | 670 (85%) | 533 (86%) |
| Hispanic | 185 (13%) | 112 (14%) | 73 (12%) |
| Unknown | 19 (1%) | 8 (1%) | 11 (2%) |
| Height, inches | 66.8 ± 4.2 | 67.2 ± 4.3 | 66.4 ± 4.1 |
| Missing data | 103 (7%) | 62 (8%) | 41 (7%) |
| Weight, pounds | 192.0 ± 1.9 | 192.1 ± 1.9 | 191.9 ± 1.9 |
| Missing data | 1,175 (84%) | 657 (83%) | 518 (84%) |
| Body mass index, kg/m^2^ | 32.7 ± 8.3 | 32.3 ± 8.2 | 33.2 ± 8.3 |
| Missing data | 430 (31%) | 248 (31%) | 182 (29%) |
|  |  |  |  |
| ***Vitals & Labs*** |  |  |  |
| Blood pressure, mm Hg |  |  |  |
| Systolic | 133 ± 20 | 132 ± 20 | 134 ± 21 |
| Diastolic | 76 ± 13 | 76 ± 13 | 76 ± 13 |
| Missing BP data | 504 (36%) | 250 (32%) | 254 (41%) |
| Total cholesterol, mg/dL | 159 ± 48 | 157 ± 49 | 161 ± 48 |
| Missing data | 605 (43%) | 363 (46%) | 242 (39%) |
| HDL-C, mg/dL | 47 ± 15 | 46 ± 14 | 48 ± 15 |
| Missing data | 619 (44%) | 369 (47%) | 250 (41%) |
| LDL-C, mg/dL | 85 ± 37 | 83 ± 35 | 87 ± 39 |
| Missing data | 617 (44%) | 366 (46%) | 251 (41%) |
| Triglyceride, mg/dL | 149 ± 88 | 147 ± 83 | 151 ± 92 |
| Missing data | 601 (43%) | 362 (46%) | 239 (39%) |
| Hemoglobin A1c, % | 7.33 ± 1.93 | 7.55 ± 2.12 | 7.06 ± 1.65 |
| Missing data | 563 (40%) | 332 (42%) | 231 (37%) |
| Serum creatinine, mg/dL | 1.31 ± 1.15 | 1.32 ± 1.23 | 1.30 ± 1.03 |
| Missing data | 150 (11%) | 98 (12%) | 52 (8%) |
| Estimated GFR, mL/min/1.73m^2^ | 52.58 ± 25.52 | 54.51 ± 26.71 | 50.22 ± 23.80 |
| Missing data | 521 (37%) | 303 (38%) | 218 (35%) |
| Serum potassium, mg/dL | 4.16 ± 0.58 | 4.21 ± 0.60 | 4.10 ± 0.55 |
| Missing data | 182 (13%) | 115 (15%) | 67 (11%) |
|  |  |  |  |
| ***Comorbidities*** |  |  |  |
| Current Smoking | 484 (34%) | 294 (37%) | 190 (31%) |
| Diabetes | 895 (64%) | 504 (64%) | 391 (63%) |
| Chronic kidney disease | 790 (56%) | 435 (55%) | 355 (58%) |
| End-stage renal disease | 26 (2%) | 16 (2%) | 10 (2%) |
| History of kidney transplant | 2 (0%) | 0 (0%) | 2 (0%) |
| Heart failure with reduced EF | 277 (20%) | 169 (21%) | 108 (18%) |
| History of CHD | 476 (34%) | 286 (36%) | 190 (31%) |
| Prior coronary revascularization | 32 (2%) | 18 (2%) | 14 (2%) |
| History of Stroke | 136 (10%) | 88 (11%) | 48 (8%) |
| History of PAD | 137 (10%) | 85 (11%) | 52 (8%) |
| History of ASCVD | 593 (42%) | 364 (46%) | 229 (37%) |
| Atrial fibrillation | 218 (15%) | 121 (15%) | 97 (16%) |
| COPD | 320 (23%) | 176 (22%) | 144 (23%) |
| Asthma | 270 (19%) | 135 (17%) | 135 (22%) |
| History of depression | 412 (29%) | 239 (30%) | 173 (28%) |
| Charlson Comorbidity Score | 6.24 ± 4.14 | 6.12 ± 4.04 | 6.39 ± 4.28 |
|  |  |  |  |
| ***Medication Use*** |  |  |  |
| Statin | 610 (43%) | 344 (44%) | 266 (43%) |
| Aspirin | 323 (23%) | 187 (24%) | 136 (22%) |
| Anticoagulants | 321 (23%) | 178 (23%) | 143 (23%) |
| *Antihypertensives* |  |  |  |
| ACE inhibitor | 790 (56%) | 790 (100%) | 0 (0%) |
| ARB | 617 (44%) | 0 (0%) | 617 (100%) |
| Direct renin inhibitor | 1 (0%) | 0 (0%) | 1 (0%) |
| Aldosterone receptor antagonist | 134 (10%) | 70 (9%) | 64 (10%) |
| Dihydropyridine CCB | 552 (39%) | 296 (37%) | 256 (41%) |
| Non-dihydropyridine CCB | 95 (7%) | 53 (7%) | 42 (7%) |
| Thiazide diuretic | 492 (35%) | 236 (30%) | 256 (41%) |
| Loop diuretic | 425 (30%) | 239 (30%) | 186 (30%) |
| Potassium-sparing diuretic | 21 (1%) | 8 (1%) | 13 (2%) |
| β-blocker | 746 (53%) | 420 (53%) | 326 (53%) |
| α_1_ blocker | 39 (3%) | 21 (3%) | 18 (3%) |
| α­_2_ agonist | 52 (4%) | 29 (4%) | 23 (4%) |
| Direct vasodilator | 177 (13%) | 96 (12%) | 81 (13%) |
|  |  |  |  |
| ***Insurance Type*** |  |  |  |
| Medicaid | 117 (8%) | 81 (10%) | 36 (6%) |
| Medicare | 392 (28%) | 226 (29%) | 166 (27%) |
| Other Government | 20 (1%) | 13 (2%) | 7 (1%) |
| Commercial Insurance or Managed Care | 173 (12%) | 102 (13%) | 71 (12%) |
| Self-pay or charity care | 22 (2%) | 10 (1%) | 12 (2%) |
| Other | 17 (1%) | 9 (1%) | 8 (1%) |
| Unknown | 47 (3%) | 30 (4%) | 17 (3%) |
| Missing data | 619 (44%) | 319 (40%) | 300 (49%) |

ACEI, angiotensin-converting enzyme inhibitor; ARB, angiotensin receptor blocker; ASCVD, atherosclerotic cardiovascular disease; CCB, calcium channel blocker; CHD, coronary heart disease; COPD, chronic obstructive pulmonary disease; EF, ejection fraction; GFR, glomerular filtration rate; HDL-C, high-density lipoprotein cholesterol; LDL-C, low-density lipoprotein cholesterol; PAD, peripheral arterial disease.

**Supplemental Table S9. Negative Control Outcomes in the Outpatient and Inpatient Cohorts.**

| **Outcome** | **ACEI/ARB- vs. non-ACEI/ARB-exposed Analysis** | | **ACEI vs. ARB-exposed Analysis** | |
| --- | --- | --- | --- | --- |
|  | **ACEI/ARB-exposed** | **Non-ACEI/ARB-exposed** | **ACEI-exposed** | **ARB-exposed** |
| ***Outpatient Cohort*** |  |  |  |  |
| *Gastrointestinal bleeding* |  |  |  |  |
| No. of events | 15 | 8 | 7 | 8 |
| Person-time^*^ | 2567 | 1236 | 1258 | 1237 |
| Rate^†^ | 0.6 | 0.6 | 0.6 | 0.6 |
| Crude HR (95% CI) | 0.94(0.40,2.22) | Ref. | 0.84(0.30,2.32) | Ref. |
| Adjusted HR (95% CI) | 1.19 (0.48, 2.98) | Ref. | 0.63(0.23,1.78) | Ref. |
| *Urinary Tract Infection* |  |  |  |  |
| No. of events | 113 | 62 | 72 | 40 |
| Person-time^*^ | 2564 | 1235 | 1256 | 1236 |
| Rate^†^ | 4.4 | 5.0 | 5.7 | 3.2 |
| Crude HR (95% CI) | 0.93(0.68,1.27) | Ref. | 1.49(1.01,2.21) | Ref. |
| Adjusted HR (95% CI) | 1.00(0.73,1.38) | Ref. | 1.31(0.89,1.93) | Ref. |
|  |  |  |  |  |
| ***Inpatient Cohort*** |  |  |  |  |
| *Gastrointestinal bleeding* |  |  |  |  |
| No. of events | 57 | 33 | 25 | 30 |
| Person-time^*^ | 569 | 284 | 303 | 244 |
| Rate^†^ | 10.0 | 11.6 | 8.2 | 12.3 |
| Crude HR (95% CI) | 0.78(0.51, 1.20) | Ref. | 0.69(0.41,1.18) | Ref. |
| Adjusted HR (95% CI) | 0.91(0.59,1.42) | Ref. | 0.70(0.41,1.19) | Ref. |
| *Urinary Tract Infection* |  |  |  |  |
| No. of events | 280 | 145 | 146 | 121 |
| Person-time^*^ | 559 | 276 | 297 | 241 |
| Rate^†^ | 50.1 | 52.5 | 49.2 | 50.3 |
| Crude HR (95% CI) | 0.85(0.70,1.04) | Ref. | 0.97(0.76,1.24) | Ref. |
| Adjusted HR (95% CI) | 0.90(0.74,1.11) | Ref. | 0.99(0.77,1.26) | Ref. |

^*^Cumulative person-years (sum of all time-to-event across all patients).

^†^No. of events divided by person-time, expressed per 100 person-years.

**Supplemental Table S10. Adjusted outcomes in the propensity score-matched sensitivity analysis.**

| **Outcome** | **ACEI/ARB-exposed vs. non-ACEI/ARB-exposed Analysis** | | **ACEI- vs. ARB-exposed Analysis** | |
| --- | --- | --- | --- | --- |
|  | **ACEI/ARB-exposed** | **non-ACEI/ARB-exposed** | **ACEI-exposed** | **ARB-exposed** |
| ***Outpatient Cohort*** |  |  |  |  |
| All-cause hospitalization or death | 0.97 (0.83, 1.15) | Ref. | 1.30 (1.10, 1.53) | Ref. |
| All-cause hospitalization | 1.01 (0.84, 1.22) | Ref. | 1.34 (1.11, 1.60) | Ref. |
| All-cause death | 0.82 (0.53, 1.27) | Ref. | 1.10 (0.73, 1.66) | Ref. |
|  |  |  |  |  |
| ***Inpatient Cohort*** |  |  |  |  |
| All-cause death | 0.75 (0.52, 1.09) | Ref. | 1.12 (0.76, 1.67) | Ref. |

Data represent adjusted hazard ratios (95% CIs) from the propensity score matched (1:1) analysis.

**Supplemental Table S11. Adjusted outcomes in the IPTW-weighted sensitivity analysis excluding individuals with compelling indications for ACEI or ARB therapy.**

| **Outcome** | **ACEI/ARB-exposed** | **non-ACEI/ARB-exposed** |
| --- | --- | --- |
| ***Outpatient Cohort*** |  |  |
| All-cause hospitalization or death | 0.97 (0.74, 1.11) | Ref. |
| All-cause hospitalization | 1.01 (0.76, 1.33) | Ref. |
| All-cause death | 0.66 (0.27, 1.61) | Ref. |
|  |  |  |
| ***Inpatient Cohort*** |  |  |
| All-cause death | 1.27 (0.31, 5.15) | Ref. |

**Supplemental Figure S1. Study Design Schematic for Outpatient COVID-19 Cohort.**

**
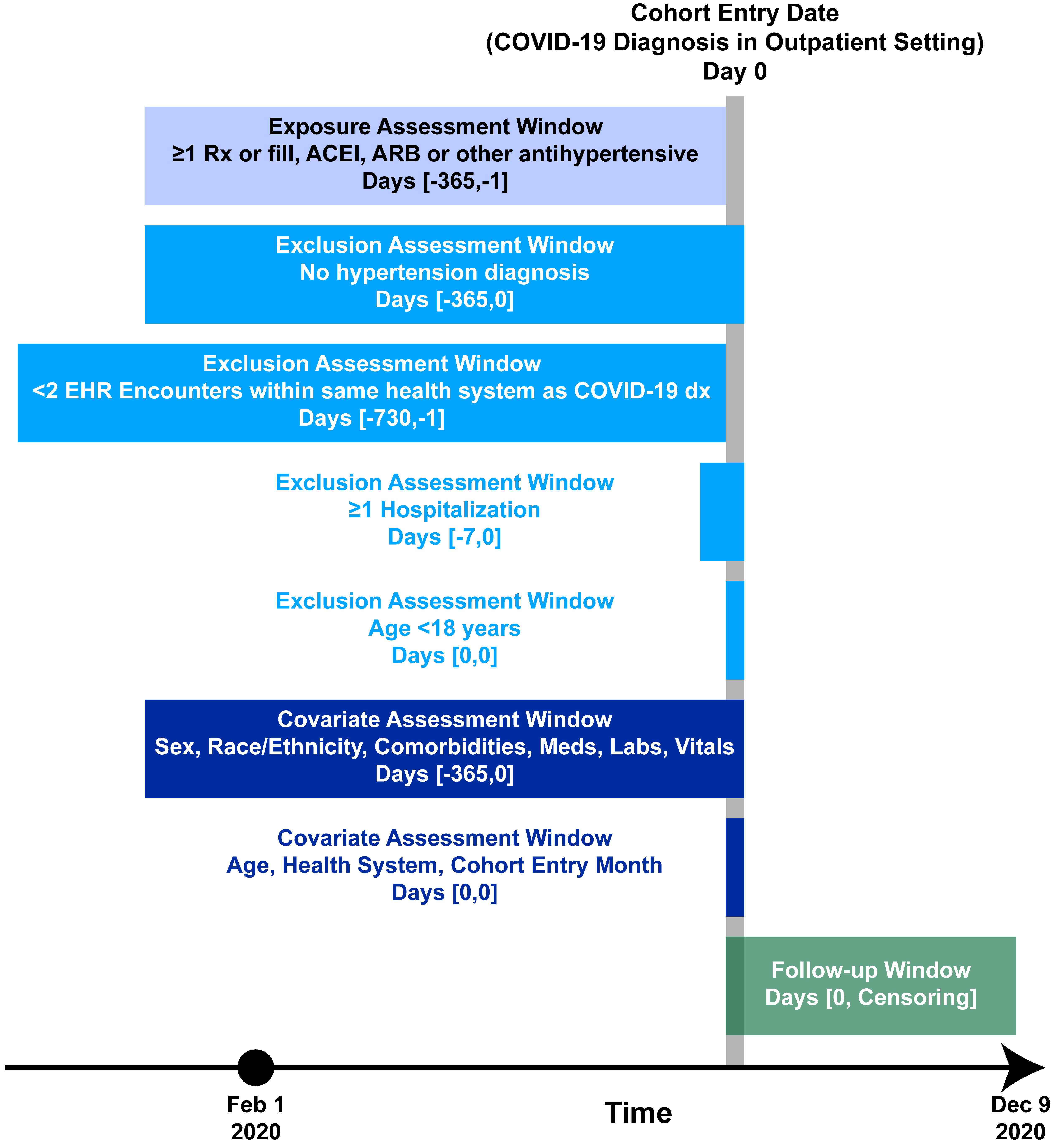
**

**Supplemental Figure S2. Study Design Schematic for Inpatient COVID-19 Cohort.** *Censoring occurred on the earliest of hospital discharge (from index hospitalization), most recent encounter date from the respective health system, or December 9, 2020.

**
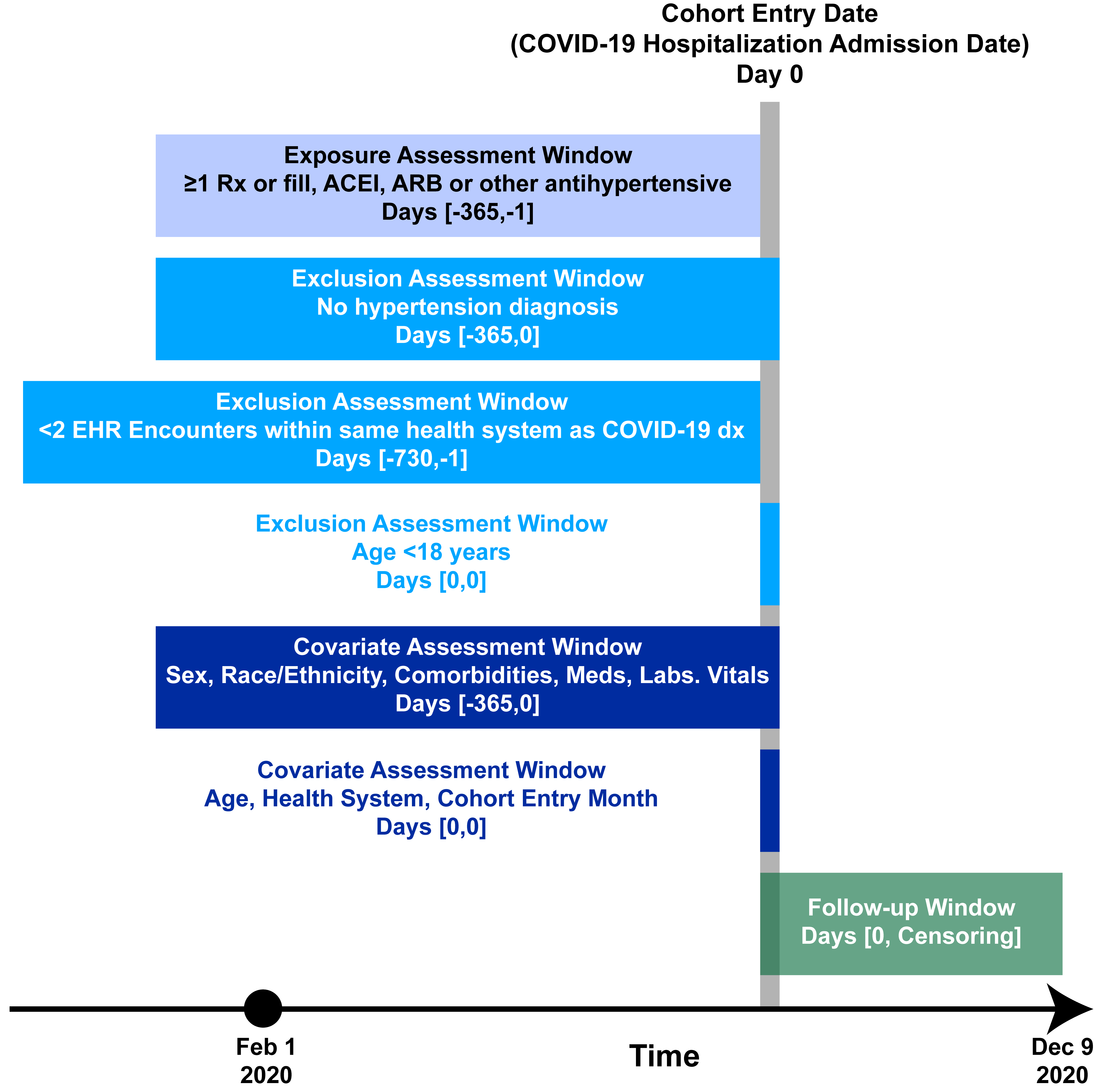
**

**Supplemental Figure S3. Flow Diagram for Development of Outpatient COVID-19 Cohort.**

**Supplemental Figure S4. Flow Diagram for Development of Inpatient COVID-19 Cohort.**

**Supplemental Figure S5. Unweighted and weighted absolute standardized mean differences for variables used in propensity score development, stratified by cohort and comparison.** Mean absolute standardized mean difference (ASMD) as circle (unweighted) or diamond (weighted), with error bars representing minimum and maximum ASMD across all imputations. The gray shaded area covers ASMDs <0.1, generally considered well-balanced.
